# Synchronized Imaging of Hydrogen Peroxide and Hydroxyl Radical in Pyroptosis and Epilepsy

**DOI:** 10.1002/advs.76679

**Published:** 2026-07-16

**Authors:** Yabing Gan, Yuling Xu, Ting Yu, Yang Li, Xin Jiang, Haoyu Jin, Haitao Li, Youyu Zhang, Peng Yin, Jong Seung Kim

**Affiliations:** ^1^ Institute of Interdisciplinary Studies Key Laboratory of Chemical Biology and Traditional Chinese Medicine Research (Ministry of Education) College of Chemistry and Chemical Engineering Hunan Normal University Changsha China; ^2^ Department of Chemistry Korea University Seoul South Korea; ^3^ National Research Laboratory for Convergence Degradation Biology Korea University Seoul South Korea

**Keywords:** epilepsy, hydrogen peroxide, hydroxyl radical, pyroptosis, synchronized imaging

## Abstract

Real‐time visualization of oxidative stress in living systems poses a formidable challenge in epilepsy research, primarily due to the scarcity of tools capable of selectively distinguishing structurally similar reactive oxygen species (ROS) with high specificity. Here, we present a dual‐functional fluorescent probe, **HH**, capable of independently detecting hydrogen peroxide (H_2_O_2_) and hydroxyl radicals (•OH) through distinct recognition mechanisms, yielding spectrally resolved signals with high sensitivity, selectivity, and biocompatibility. **HH** enables high‐resolution, interference‐free imaging of redox dynamics in both gasdermin‐mediated pyroptosis and pentylenetetrazol (PTZ)‐induced epileptic cell models. Furthermore, **HH** was successfully employed in PTZ‐induced zebrafish and kainic acid (KA)‐induced mouse epilepsy models, revealing elevated ROS levels under seizure‐like conditions. Notably, ROS‐targeted therapeutic interventions significantly modulated oxidative profiles, demonstrating **HH**’s potential for therapeutic evaluation. This work not only fills a critical methodological gap in ROS biology and epilepsy research but also provides a versatile platform for studying redox regulation in neurological and inflammatory diseases and enabling mechanistic and therapeutic investigations.

## Graphical abstract

A dual‐responsive fluorescent probe, HH, enables synchronized visualization of H_2_O_2_ and •OH through spectrally resolved green and red channels. By tracking redox dynamics in pyroptosis and epilepsy models, HH reveals ROS elevation during seizure‐like pathology and supports evaluation of antioxidant and antiepileptic therapeutic interventions.

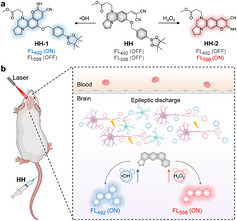

## 1 Introduction

Epilepsy is one of the most common and refractory neurological disorders, affecting over 70 million people worldwide, and is characterized by recurrent, spontaneous seizures that severely impair quality of life and increase the risk of neurodegeneration, psychiatric comorbidities, and sudden death [1, 2]. Despite intensive research, the molecular pathogenesis of epilepsy remains elusive due to its heterogeneous etiology and multifactorial nature. In recent years, increasing evidence has implicated gasdermin‐mediated pyroptosis, a pro‐inflammatory form of programmed cell death, in the initiation and progression of epilepsy and other central nervous system (CNS) pathologies such as ischemic stroke and traumatic brain injury [3]. A key driver of this pathological axis is oxidative stress, wherein reactive oxygen species (ROS), particularly hydrogen peroxide (H_2_O_2_) and hydroxyl radicals (•OH), play pivotal roles in triggering neuroinflammation, mitochondrial dysfunction, and neuronal loss [4, 5, 6]. H_2_O_2_ is a relatively stable and diffusible ROS that functions as a second messenger in redox signaling and cellular homeostasis under physiological conditions [7, 8, 9]. However, under pathological stress, its excessive accumulation disrupts redox equilibrium, promotes lipid peroxidation, and renders neurons more susceptible to damage [10, 11, 12]. Critically, H_2_O_2_ participates in the Fenton reaction, producing •OH—one of the most reactive and cytotoxic ROS—which indiscriminately oxidizes biomolecules including nucleic acids, lipids, and proteins. Growing evidence implicates •OH in inducing irreversible oxidative damage and exacerbates seizure activity, as well as possibly initiates epileptogenesis [13]. Despite the biological significance of H_2_O_2_ and •OH, their real‐time, spatially resolved detection in living systems remains technically challenging. In particular, the spatiotemporal interplay between H_2_O_2_ and •OH in pyroptosis and epileptic pathology remains poorly understood, predominantly due to the scarcity of suitable chemical tools that enable simultaneous, selective, and real‐time monitoring in complex biological milieus.

**FIGURE 1:**
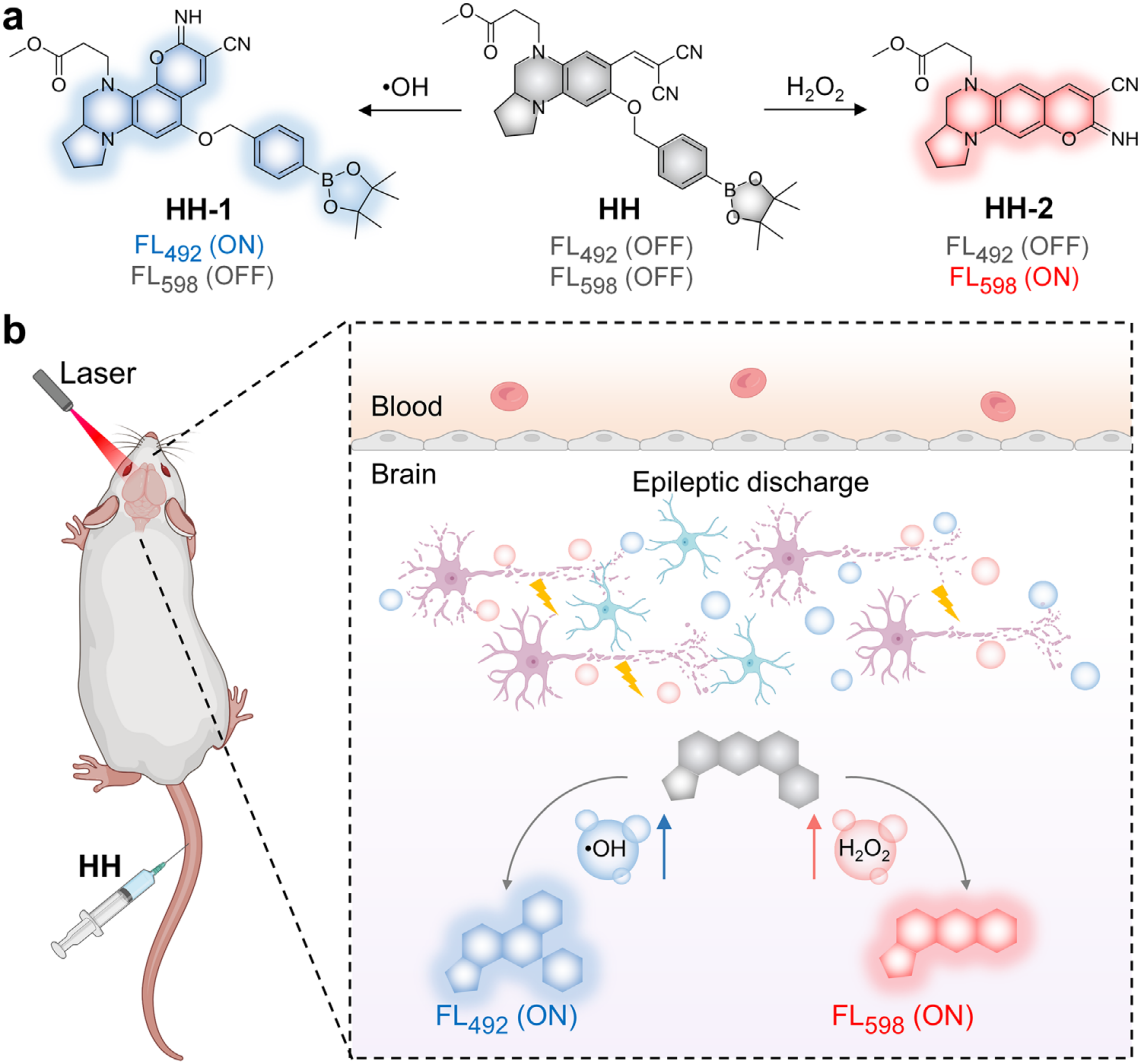
a) The working mechanism of **HH** for simultaneous detection of H_2_O_2_ and •OH. b) Schematic illustration for monitoring H_2_O_2_ and •OH levels in kainic acid (KA)‐induced epilepsy models.

Fluorescent probes have emerged as indispensable tools in biomedical research, offering non‐invasive, high‐sensitivity, and real‐time capabilities for monitoring molecular dynamics in living systems [14, 15, 16]. A range of ROS‐responsive fluorescent probes have been reported for applications in epilepsy [17, 18, 19, 20, 21, 22, 23, 24, 25, 26, 27, 28, 29, 30, 31, 32] and redox biology (Table S1), including dual‐mode probes for superoxide (O_2_^•^^−^) [33], near‐infrared (NIR) viscosity‐sensitive probes for peroxynitrite (ONOO^−^) [34], and ratiometric probes for H_2_O_2_ in pyroptotic pathways [35]. However, these approaches often rely on single‐analyte detection or multi‐probe combinations, which suffer from poor cellular co‐localization, inconsistent pharmacokinetics, and overlapping signal outputs—factors that compromise data fidelity and ultimately limit utility in dynamic redox biology [36]. The multi‐channel fluorescent probes developed in recent years integrate multiple specific recognition sites within a unified molecular framework, allowing simultaneous monitoring of diverse biomolecules through spectrally independent fluorescence channels. Such probes not only effectively circumvents shortcomings of multi‐probe combinations but also provides a high‐fidelity, multi‐dimensional real‐time molecular information platform for early disease diagnosis, dynamic disease progression tracking, and targeted therapy evaluation [37]. Although some multichannel or multifunctional fluorescent probes for simultaneous detection of dual ROS have been reported (Table S2), most of these dual‐channel probes are primarily focused on other ROS pairs, such as HOCl/ONOO^−^ or H_2_O_2_/HOCl. To date, a single dual‐channel fluorescent probe capable of simultaneously imaging both H_2_O_2_ and •OH has not yet been reported. To overcome these limitations, we rationally design a dual‐functional fluorescent probe (named **HH**, Figure 1) that integrates two orthogonal recognition motifs: an aryl boronic ester for selective detection of H_2_O_2_ and a hydroxylation aromatic unit responsive to •OH. Upon reaction, **HH** generates two structurally distinct coumarin derivatives, each exhibiting independent, non‐overlapping fluorescence emissions, enabling simultaneous, interference‐free detection of H_2_O_2_ and •OH with high spatiotemporal resolution in live cells and animals.

In this study, we validate the biological utility of probe **HH** in a series of in vitro and in vivo models. We first monitored ROS dynamics during gasdermin‐mediated pyroptosis, revealing distinct oxidative signatures associated with cell death. We then applied the probe in two widely used epilepsy models—pentylenetetrazol (PTZ)‐induced zebrafish and kainic acid (KA)‐induced mouse models—to visualize the real‐time oxidative surge of H_2_O_2_ and •OH during seizure progression. Notably, we demonstrate that therapeutic modulation of redox homeostasis alters probe readouts, highlighting its potential as a molecular tool for treatment evaluation and mechanistic exploration. Overall, this work not only addresses a critical technical barrier in epilepsy and redox biology but also lays a foundation for broader applications in neuroinflammation, neurodegeneration, and oxidative disease research.

## 2 Results and Discussion

### 2.1 Design and Synthesis of a Dual‐Responsive Fluorescent Probe

To enable the simultaneous detection of H_2_O_2_ and •OH, we rationally designed a unimolecular dual‐responsive fluorescent probe, as illustrated in Figure 1. Coumarin derivatives were chosen as the fluorophore backbone owing to their excellent photostability, high quantum yield, and favorable biocompatibility [38, 39]. To further enhance optical performance, a tetrahydroquinoxaline moiety and a five‐membered pyrrolidine ring were introduced to the coumarin core, thereby increasing the Stokes shift and overall fluorescence brightness [40, 41, 42, 43, 44]. In addition, a methyl ester group was incorporated to improve water solubility and lipophilicity (with a measured log*P* of 1.69), which could facilitate improved cellular permeability and imaging performance [45]. During the synthesis of intermediate compound 1‐A (Scheme S1), we observed significant instability and susceptibility to oxidative degradation. Notably, the C‐3 position (highlighted in compound 1‐A) was prone to electrophilic addition, consistent with previous findings reported by Xian et al. [46]. Drawing inspiration from hydroxyl radical‐selective probe based on electrophilic addition chemistry reported by Ma et al. (compound 1‐C in Scheme S1) [47, 48], we hypothesized that compound 1‐B might react with •OH via aromatic hydroxylation, followed by intramolecular cyclization with the adjacent cyano group to form a fluorescent coumarin derivative.

Conventionally, coumarin‐based probes (compound 1‐D in Scheme S1) relied on phenolic hydroxyl‐protected recognition units that, upon analyte interaction, undergo cyanide‐mediated cyclization to generate strong fluorescent signals. In our design, the incorporation of both tetrahydroquinoxaline and pyrrolidine units allowed for tunable intramolecular charge transfer (ICT) characteristics across the resulting coumarin derivatives [49]. This structural divergence yielded two distinct emission maxima, allowing for spectrally resolvable dual‐channel fluorescence. As depicted in Figure 1a, the final probe, named **HH**, integrated two orthogonal recognition mechanisms: (i) •OH‐triggered aromatic hydroxylation and subsequent cyclization to generate **HH‐1**, characterized by bright green fluorescence; and (ii) H_2_O_2_‐induced oxidative deprotection of the boronate ester, followed by cyclization to form **HH‐2**, which emitted strong red fluorescence. Remarkably, the fluorescence emissions of **HH‐1** and **HH‐2** were separated by a substantial wavelength interval of 106 nm, effectively minimizing spectral crosstalk and enabling the simultaneous and spectrally resolved detection of H_2_O_2_ and •OH. The complete synthetic route of **HH** and its derivatives was presented in Schemes S2 and S3. All intermediates and final products were thoroughly characterized using ^1^H NMR, ^13^C NMR, IR spectroscopy, and high‐resolution mass spectrometry (HRMS) (see Figures S27–S43)

### 2.2 In Vitro Characterization

**FIGURE 2:**
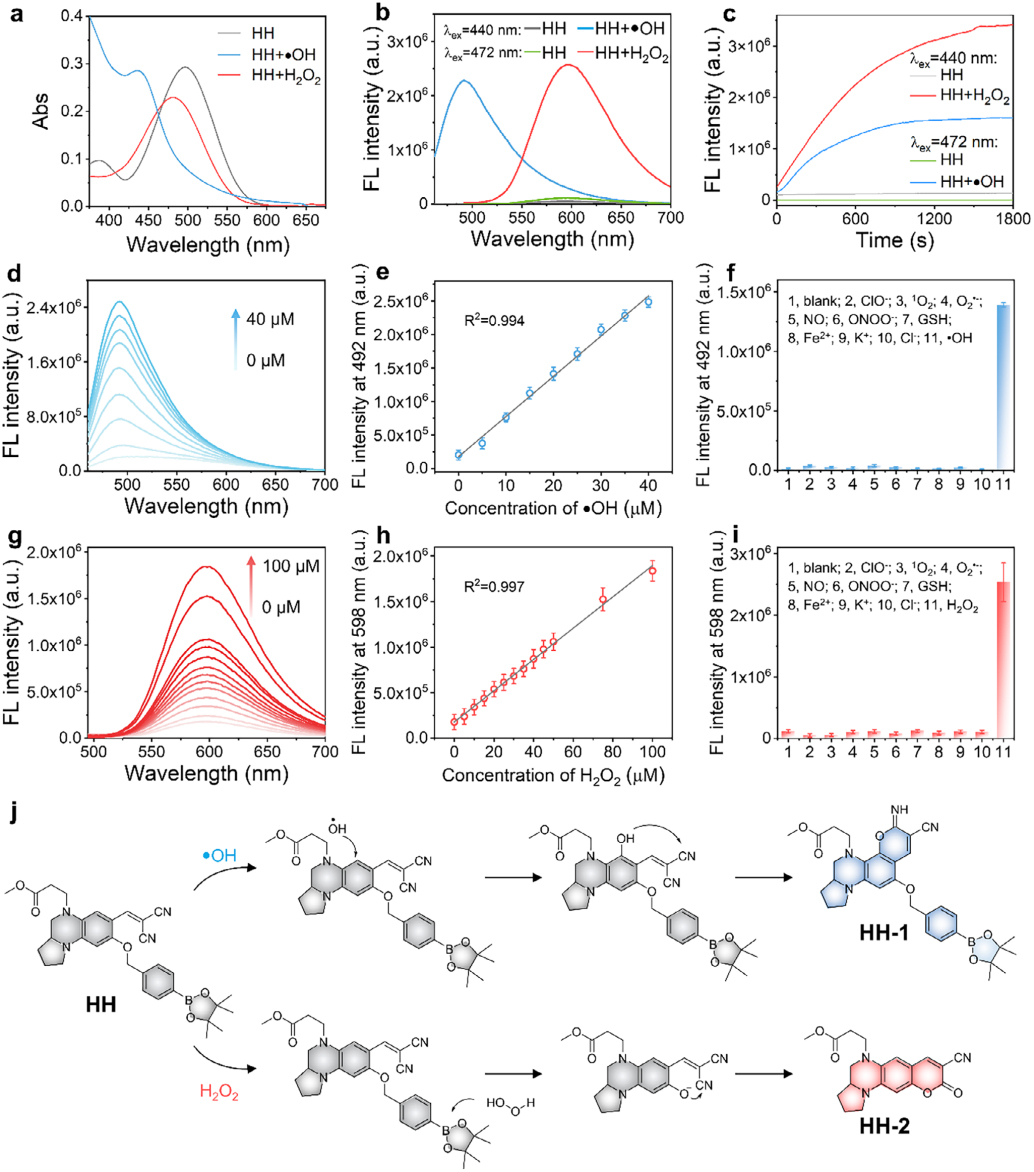
UV–vis absorption (a) and fluorescence emission (b) spectra of **HH** (10 µm) upon the addition of •OH (10 equiv.) or H_2_O_2_ (10 equiv.) in EtOH/PBS buffer (v/v = 2:8) at r.t. for 30 min. (c) The corresponding time‐dependent fluorescence intensity changes of **HH** upon the addition of •OH (30 equiv.) and H_2_O_2_ (30 equiv.) monitored at 492 and 598 nm. (d, g) Fluorescence intensity spectra of **HH** (10 µm) upon titration of •OH/H_2_O_2_ in EtOH/PBS buffer (v/v = 2:8). (e, h) Corresponding linear calibration plots of fluorescence intensity of **HH** at 492 nm (e) and 598 nm (h) as a function of •OH and H_2_O_2_ concentration, respectively. (f, i) Selectivity of **HH** (10 µm) toward •OH (f) and H_2_O_2_ (i) over other biologically relevant species including 1, blank; 2, ClO^−^; 3, ^1^O_2_; 4, O_2_^•−^; 5, NO; 6, ONOO^−^; 7, GSH; 8, Fe^2+^; 9, K^+^; 10, Cl^−^; 11, •OH in (f) and H_2_O_2_ in (**i**); all at 100 µm. (j) Proposed sensing mechanism of **HH** toward •OH and H_2_O_2_, forming fluorescent products **HH‐1** and **HH‐2**, respectively. *λ*_ex_ = 440 nm for •OH and 472 nm for H_2_O_2_.

With the dual‐responsive probe **HH** in hand, we systematically evaluated its optical behavior toward H_2_O_2_ and •OH under physiologically relevant conditions. Spectroscopic measurements were carried out in EtOH/PBS buffer (v/v = 2:8) at ambient temperature. Upon the addition of H_2_O_2_, the maximum absorption peak of **HH** exhibited a slight blue shift from 496 nm to 470 nm, accompanied by a pronounced fluorescence enhancement (21‐fold, *Φ* = 0.05, Figure S1) at 598 nm when excited at 472 nm. In contrast, treatment with •OH caused a significant hypsochromic shift in the absorption maximum from 498 nm to 440 nm (Figure 2a). Fluorescence measurements revealed a strong turn‐on signal at 492 nm (*λ*_ex_ = 440 nm), with over 180‐fold intensity enhancement (*Φ* = 0.09) (Figure 2b). Notably, the emission maxima for the H_2_O_2_‐ and •OH‐responsive products were well separated by more than 100 nm, allowing for unambiguous discrimination in dual fluorescence channels with minimal spectral overlap.

We next examined the reaction kinetics of **HH** with H_2_O_2_ and •OH. As depicted in Figure 2c, the reaction with •OH reached fluorescence saturation within 15 min, and the H_2_O_2_‐triggered response plateaued at 30 min. Furthermore, after responding to H_2_O_2_ and •OH, the probe still maintains excellent photostability under continuous excitation (Figure S6). To confirm dual responsiveness of H_2_O_2_ and •OH level, **HH** was first incubated with H_2_O_2_ (10 equiv.), followed by graded additions of Fe^2^^+^ to generate •OH in situ. This led to simultaneous emergence of green and red fluorescence signals (*λ*_ex_ = 440 nm), with increasing green fluorescence intensity proportional to Fe^2^^+^ concentration (Figure S2). On the other hand, after reacting the probe with a small amount of pre‐prepared •OH, the subsequent addition of different concentrations of H_2_O_2_ to the system also allowed the detection of dual‐emission behavior at 492 nm and 598 nm. These findings strongly support that **HH** is capable of independently detecting H_2_O_2_ and •OH under coexisting conditions through two distinct emission channels without cross‐interference. As shown in Figure S3, after the probe has fully reacted with either H_2_O_2_ or •OH, subsequent addition of the other analyte does not induce any further change in the fluorescence signal. This indicates that the two reactive sites of the probe are independent and that no sequential cross‐reactivity occurs.

To assess sensitivity of dual‐responsive probe **HH**, we performed dose‐response experiments for each ROS. As shown in Figure 2d, increasing •OH concentrations led to a proportional rise in fluorescence at 492 nm, achieving ≈180‐fold enhancement at 4 equivalents. The response showed excellent linearity over 0–40 µm (*R* ^2^ = 0.9936) with a calculated limits of detection (LOD) of 4.9 nm (Figure 2e). Similarly, H_2_O_2_ induced a strong dose‐dependent fluorescence enhancement at 598 nm, linear over the range of 0–100 µm (*R* ^2^ = 0.9960), with a LOD of 18.6 nm (Figure 2g,h). These data confirm the high sensitivity of **HH** for both H_2_O_2_ and •OH, with detection limits suitable for tracking endogenous ROS fluctuations in biological samples. Selectivity studies were conducted against a broad panel of biologically relevant species, including reactive oxygen, nitrogen, and sulfur species (ROS/RNS/RSS), metal ions (Al^3+^, Fe^2+^, Zn^2+^, K^+^, Ni^2+^, Cu^2+^), halides, and amino acids (Glu, Lys, Try, Gly, L‐Thr, His). Only •OH and H_2_O_2_ triggered significant fluorescence responses, while all other analytes (ClO^−^, ^1^O_2_, O_2_^•−^, TBHP, NO, ONOO^−^, Cys, Hcy, GSH, NAC, Na_2_SO_3_, Na_2_S, Al^3+^, Fe^2+^, Zn^2+^, K^+^, Ni^2+^, Cu^2+^, F^−^, I^−^, Cl^−^, Br^−^, Glu, Lys, Try) produced negligible changes (Figure 2f,i and Figures S4 and S5). Furthermore, under physiological conditions (Figure S7), the probe maintains good response performance toward H_2_O_2_ and •OH. These studies specificity validated **HH** as a robust dual‐emission probe capable of separate, simultaneous, sensitive, and selective detection of H_2_O_2_ and •OH, establishing its strong potential for real‐time visualization of ROS dynamics in live‐cell and in vivo models.

### 2.3 Response Mechanism for Detecting •OH and H_2_O_2_

To elucidate the underlying mechanisms by which probe **HH** responded to •OH and H_2_O_2_, we employed both experimental verification and structural analog comparison strategies. Initially, direct isolation and structural characterization of the •OH‐induced reaction product proved technically challenging, likely due to the high reactivity and short‐lived nature of •OH. As an alternative approach, we synthesized a series of model coumarin derivatives (compounds 2–6) via analogous reaction pathways (Scheme S3), and fully characterized them using both ^1^H and ^13^C NMR spectroscopy (see Figures S31–S39). These model compounds differed from the proposed •OH‐derived product **HH‐1** only in the presence or absence of a substituent at the C5 position—a variation previously reported to have negligible influence on the photophysical properties of coumarin fluorophores. Spectroscopic comparisons showed that **HH** treated with •OH in EtOH/PBS (v/v = 2:8) exhibited absorption and emission profiles nearly identical to those of compounds 2–6 (*λ*_abs_ ≈ 438/432 nm; *λ*_em_ ≈ 492/498 nm; Figure S8). These results strongly supported the proposed •OH‐triggered aromatic hydroxylation followed by intramolecular cyclization leading to **HH‐1**, as depicted in Figure 2j and Scheme S4. Furthermore, High‐resolution mass spectrometry (HRMS) was utilized to further confirm the response mechanism. Upon addition of •OH into **HH** solution, an ion peak (m/z) at 585.2862 was found in HRMS, which was consistent with the MS of the expected product **HH‐1** (calculated for 585.2879, Figure S10).

For the H_2_O_2_ response mechanism, we successfully isolated the oxidation product formed upon treatment of **HH** with H_2_O_2_. UV–vis and fluorescence analyses demonstrated that the isolated product matched compound **HH‐2**, as evidenced by overlapping absorption and emission profiles (Figure S9). In addition, HRMS further corroborated the reaction mechanism, with the detected ion peak matching the theoretical value calculated for **HH‐2** (calculated for 363.1608, Figure S11). These correspondence verified the proposed H_2_O_2_‐responsive mechanism involving oxidative deprotection of the boronate ester and subsequent cyclization to generate a red‐emissive coumarin derivative. Taken together, these findings confirmed that probe **HH** could function via two orthogonal and well‐defined chemical pathways for selective detection of •OH and H_2_O_2_, each yielding a distinct fluorogenic signature with minimal spectral crosstalk.

### 2.4 Dual‐Channel Cellular Imaging

**FIGURE 3:**
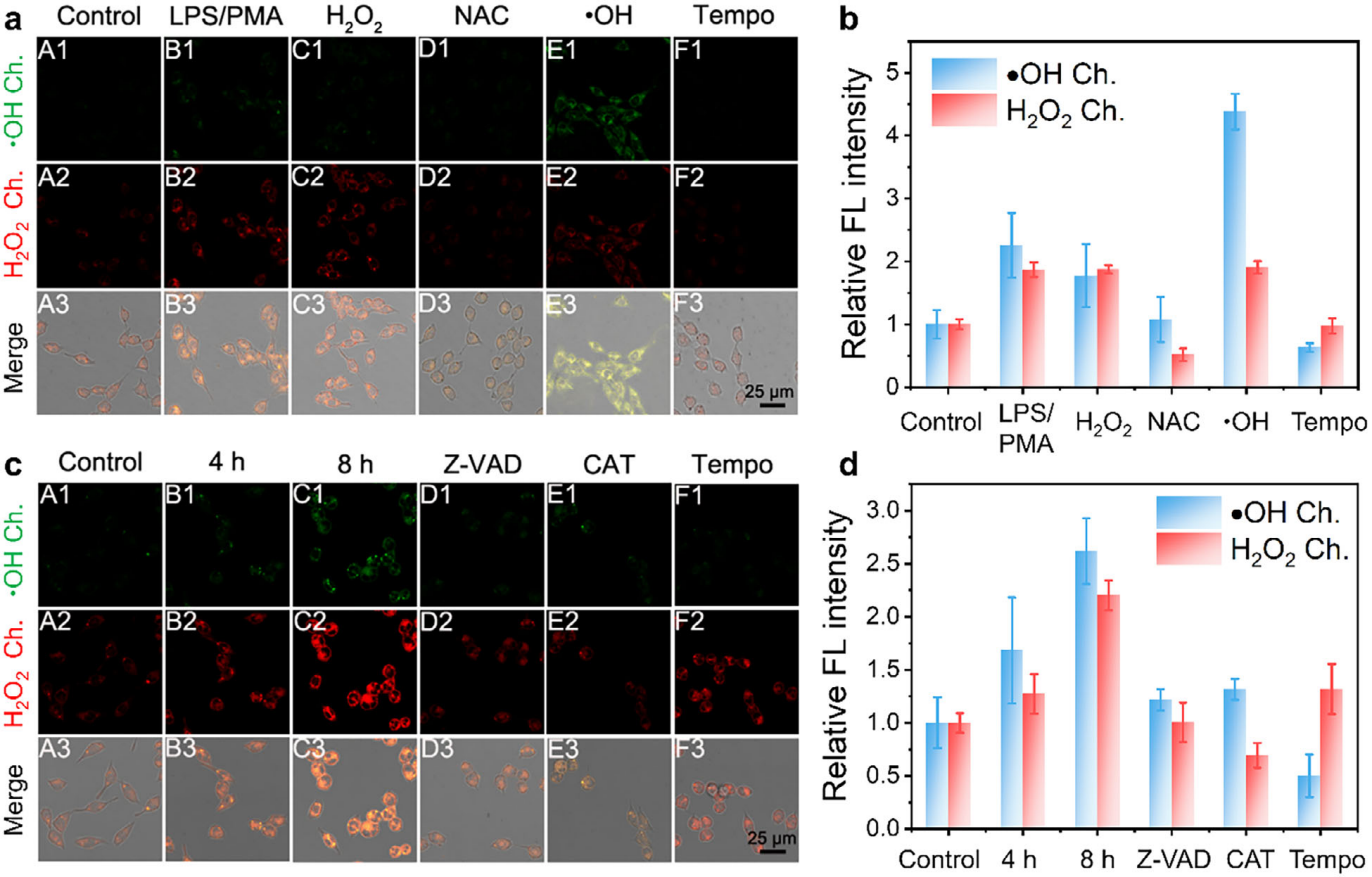
(a,b) Confocal fluorescence imaging of endogenous and exogenous •OH and H_2_O_2_ in HeLa cells. (A1–A3) Cells were incubated with probe **HH** (5 µm) for 30 min. In the other groups, the cells were pretreated with LPS/PMA (1 µg mL^−^^1^) (B1–B3) /TEMPO (0.1 mm) (F1–F3) and NAC (0.25 mm) (D1‐D3) for 30 min, and then incubated with probe **HH** (5 µm, 30 min). Cells were pretreated with TEMPO (0.1 mm)/ NAC (0.25 mm) for 30 min, subsequently incubated with probe **HH** (5 µm, 30 min), and finally incubated with •OH (100 µm) (E1‐E3) / H_2_O_2_ (100 µm) (C1–C3) for 30 min. (c, d) Monitoring of •OH and H_2_O_2_ generation in pyroptosis induced by DM‐αKG. (A1–C3) Cells pretreated with 15 mm DM‐αKG for 0, 4, or 8 h, and then incubated with 5 µm probe **HH** for 30 min. (D1–F3) Cells pretreated with 15 mm DM‐αKG in the presence of (D1–D3) 50 µm Z‐VAD‐FMK, (E1–E3) 100 µm CAT or (F1–F3) 100 µm TEMPO for 8 h, and then incubated with 5 µm probe **HH** for 30 min. Green channel for •OH: *λ*_ex_ = 458 nm, *λ*_em_ = 480–560 nm; red channel for H_2_O_2_: *λ*_ex_ = 476 nm, *λ*_em_ = 580–650 nm. Scale bar: 25 µm.

Encouraged by the favorable photophysical properties and excellent selectivity of probe **HH** for H_2_O_2_ and •OH in vitro, we next evaluated its potential for live‐cell imaging. Cytotoxicity was first assessed via MTT assays in HeLa and SH‐SY5Y cell lines. The results showed that probe **HH** exhibited negligible cytotoxicity at concentrations up to 50 µm (Figure S12), confirming its biocompatibility for live‐cell applications. Given its high sensitivity, selectivity, and low toxicity, we hypothesized that probe **HH** could enable the dual‐channel imaging of intracellular H_2_O_2_ and •OH. HeLa and SH‐SY5Y cells were selected as representative models. When incubated with **HH** (5 µm) alone, HeLa cells exhibited weak fluorescence in both the green (480–560 nm) and red (580–650 nm) channels (Figure 3a. A1–A2). Upon co‐treatment with H_2_O_2_ or •OH (100 µm), a significant enhancement in red and green fluorescence signals was observed, respectively (Figure 3a. C1–C3 and E1–E3), confirming **HH**’s responsiveness to exogenous ROS. To further verify the specificity of each fluorescence channel, cells were pretreated with the corresponding ROS scavengers before ROS stimulation. Pretreatment with N‐acetyl‐L‐cysteine (NAC, 0.25 mm), a broad‐spectrum antioxidant, attenuated the H_2_O_2_‐induced red fluorescence response (Figure 3a, D1–D3), whereas pretreatment with 2,2,6,6‐tetramethyl‐1‐piperidinyloxy (TEMPO, 0.1 mm), a hydroxyl radical scavenger, significantly attenuated the green fluorescence elicited by •OH (Figure 3a, F1–F3). These observations are fully consistent with the expected functions of NAC and TEMPO, further supporting the selective assignment of the red and green fluorescence channels to H_2_O_2_ and •OH, respectively. Furthermore, stimulation with lipopolysaccharide (LPS, 1 µg mL^−^^1^) and phorbol 12‐myristate 13‐acetate (PMA, 1 µg mL^−^^1^) significantly enhanced fluorescence in both channels (Figure 3a, B1–B2), indicating the simultaneous elevation of intracellular H_2_O_2_ and •OH during oxidative stress. Consistent results were also observed in SH‐SY5Y neuronal cells and zebrafish (Figures S13 and S14), demonstrating **HH**’s robust dual‐channel imaging capability across different biological systems. To further validate the selectivity of **HH**, we systematically evaluated its imaging responses toward other reactive oxygen species (ROS) (Figure S15) and performed additional mechanistic control experiments using the •OH inducer cinnamaldehyde, the •OH scavengers mannitol and DMSO, and the H_2_O_2_ scavenger catalase (Figures S16–S18). Treatment with cinnamaldehyde resulted in a pronounced enhancement of the green fluorescence signal, accompanied by only a slight increase in the red fluorescence signal, indicating the preferential generation of •OH. In contrast, pretreatment with the •OH scavengers mannitol or DMSO markedly attenuated the green fluorescence while exerting minimal influence on the red fluorescence channel, confirming that the green‐channel response originates predominantly from intracellular •OH. Likewise, catalase, which selectively scavenges H_2_O_2_, significantly reduced the red fluorescence signal with little effect on the green channel, verifying that the red‐channel fluorescence specifically corresponds to H_2_O_2_. Collectively, these results demonstrate that both fluorescence channels respond selectively to their respective ROS species and that the corresponding scavengers effectively suppress the fluorescence changes in the expected channels, thereby confirming the excellent specificity of **HH** for the discriminative imaging of H_2_O_2_ and •OH.

Encouraged by its performance in cellular assays, we further investigated whether **HH** could monitor ROS dynamics during pyroptosis. Dimethyl‐α‐ketoglutarate (DM‐αKG) was used to induce pyroptosis in HeLa cells, a model previously reported to activate gasdermin‐mediated pathways [50]. As shown in Figures S19 and S20, DM‐αKG induced typical pyroptotic features, including cell swelling, membrane blebbing, and rupture, which were blocked by the pan‐caspase inhibitor Z‐VAD‐FMK (50 µm), consistent with a previously reported DM‐αKG‐induced pyroptotic cell death model [51]. Using probe **HH**, we simultaneously monitored intracellular H_2_O_2_ and •OH levels during pyroptosis. Compared with the control group, treatment with DM‐αKG for 4 or 8 h resulted in a time‐dependent increase in fluorescence intensity, reaching 1.7‐ and 2.6‐fold in the green channel (•OH) and 1.2‐ and 2.2‐fold in the red channel (H_2_O_2_), respectively (Figure 3b, B1–C3 and Figure S21). To further verify that these fluorescence changes originated from pyroptosis‐associated ROS generation, cells were pretreated with Z‐VAD‐FMK, catalase (CAT), or TEMPO prior to **HH** imaging. Z‐VAD‐FMK, a pan‐caspase inhibitor that suppresses pyroptosis, markedly reduced fluorescence in both channels, consistent with diminished intracellular ROS production during inhibition of pyroptotic progression. CAT selectively scavenges H_2_O_2_ and therefore primarily attenuated the red fluorescence signal, whereas TEMPO scavenges hydroxyl radicals and predominantly reduced the green fluorescence signal. The fluorescence changes observed after these treatments were in excellent agreement with the expected biological functions of each reagent, further supporting the assignment of the red and green channels to H_2_O_2_ and •OH, respectively. Collectively, these results demonstrate that **HH** is an effective tool for real‐time visualization of intracellular ROS dynamics and reveal that the accumulation of both H_2_O_2_ and •OH is closely associated with the progression of pyroptosis.

### 2.5 Monitoring •OH and H_2_O_2_ Dynamics and Evaluating Therapeutic Intervention in Living Cell Epilepsy Models

Epilepsy is a chronic neurological disorder that poses a significant threat to global health and is increasingly associated with ROS‐mediated oxidative stress [52]. Despite extensive studies, the molecular mechanisms linking ROS to seizure activity remain insufficiently defined, particularly regarding the interplay between H_2_O_2_ and •OH during epileptogenesis. To this end, we employed our dual‐functional probe **HH** to spatiotemporally monitor intracellular ROS fluctuations during seizure‐like conditions and assess therapeutic interventions.

**FIGURE 4:**
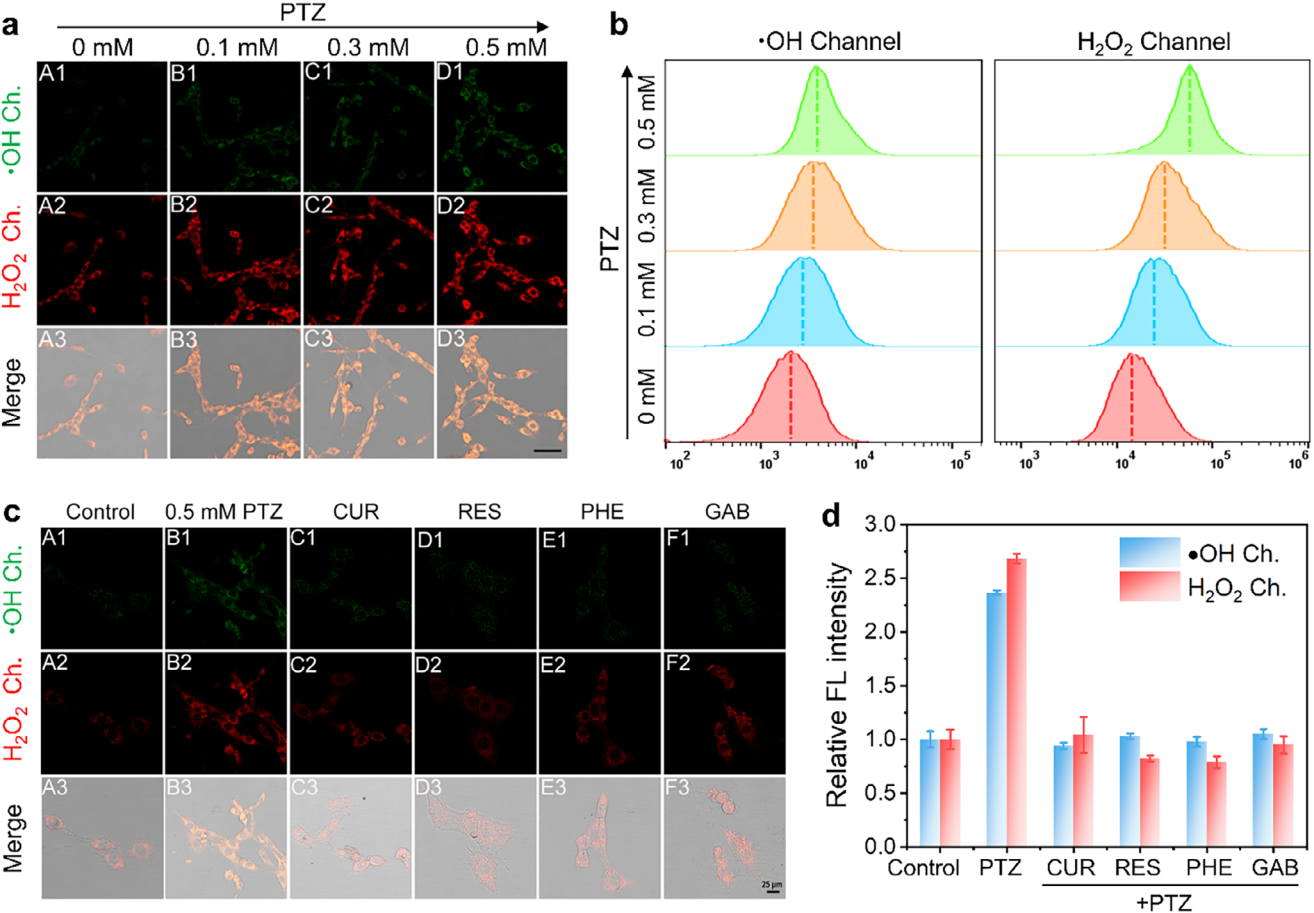
Dual‐channel detection of •OH and H_2_O_2_ in PTZ‐induced epilepsy models of SH‐SY5Y cells. (a) Confocal fluorescence images of •OH (green) and H_2_O_2_ (red) levels variations in cells treated with increasing concentrations of PTZ for 8 h, followed by incubation with **HH** (5 µm) for 30 min. Scale bar: 50 µm. (b) Flow cytometric analysis of ROS levels in cells treated as in (a), showing log‐scale fluorescence intensity distributions for •OH (left) and H_2_O_2_ (right) channels. (c) Representative confocal images of cells pretreated with PTZ (0.5 mm, 8 h), followed by treatment with CUR (30 µm), RES (30 µm), PHE (30 µm), or GAB (1 mm) for 2 h, and then stained with **HH** (5 µm, 30 min). Fluorescence from •OH (green) and H_2_O_2_ (red) channels is shown separately and merged. Scale bar: 50 µm. (d) Quantification of relative fluorescence intensities from both channels in panel (c). Data are normalized to the control group and presented as mean ± SD (*n* = 3).

We first established a cellular model of epilepsy by treating SH‐SY5Y neuroblastoma cells with pentylenetetrazole (PTZ), a well‐characterized GABA receptor antagonist known to induce neuronal hyperexcitability [53]. Cells were treated with increasing PTZ concentrations (0, 0.1, 0.3, and 0.5 mm) for 8 h, followed by incubation with **HH** (5 µm) for 30 min. As shown in Figure 4a, the fluorescence intensities corresponding to H_2_O_2_ (red channel) and •OH (green channel) increased proportionally with PTZ concentration. This observation was corroborated by flow cytometric analysis (Figure 4b), which revealed a consistent dose‐dependent elevation of ROS levels. These findings suggested that intracellular accumulation of H_2_O_2_ and •OH is strongly associated with PTZ‐induced seizure‐like injury, suggesting their involvement in oxidative stress‐related neuronal damage.

To evaluate therapeutic modulation of oxidative stress, we next investigated the effects of representative neuroprotective, antioxidant, and antiepileptic drugs. After pre‐treatment with PTZ (0.5 mm) for 8 h, cells were subsequently incubated to curcumin (CUR, 30 µm), resveratrol (RES, 30 µm), phenytoin sodium (PHE, 30 µm), or gabapentin (GAB, 1 mm) for 2 h, followed by staining with **HH** (5 µm). Fluorescence imaging (Figure 4c) and relevant semi‐quantitative analysis (Figure 4d) revealed significant reductions in H_2_O_2_ and •OH levels across all drug‐treated groups, with varying degrees of efficacy. These results indicated that the observed antiepileptic actions of the tested agents could be partially attributable to ROS scavenging or oxidative pathways modulation. Collectively, these experiments demonstrate that probe **HH** enables real‐time visualization of oxidative dynamics in seizure‐like cellular models and suggest its potential utility for identifying and evaluating candidate antiepileptic therapies. These findings further support the close association between oxidative stress and seizure‐like neuronal injury.

### 2.6 Real‐Time Monitoring in Animal Models

Building upon the promising results of the in vitro cell imaging studies, we next sought to evaluate the in vivo applicability of probe **HH** for monitoring endogenous fluctuations of H_2_O_2_ and •OH during epilepsy‐like conditions. To this end, we established two widely recognized epilepsy models: a chronic epilepsy model in zebrafish larvae induced by pentylenetetrazol (PTZ) [54], and an acute epilepsy model in mice induced by kainic acid (KA) [55]. Zebrafish at 8 d post‐fertilization (dpf), a developmental stage characterized by a mature nervous system and high optical transparency, were selected for imaging studies. The larvae were incubated with PTZ (6 mm) for varying durations to induce chronic seizure‐like activity, followed by exposure to probe **HH** (10 µm) for 30 min. Confocal imaging results showed that the fluorescence signals of both the red channel (H_2_O_2_) and the green channel (•OH) in the zebrafish brain gradually increased with prolonged PTZ exposure time (Figure S22) and were significantly higher than those in the untreated control group, indicating dynamic fluctuations of reactive oxygen species under seizure‐like conditions.

**FIGURE 5:**
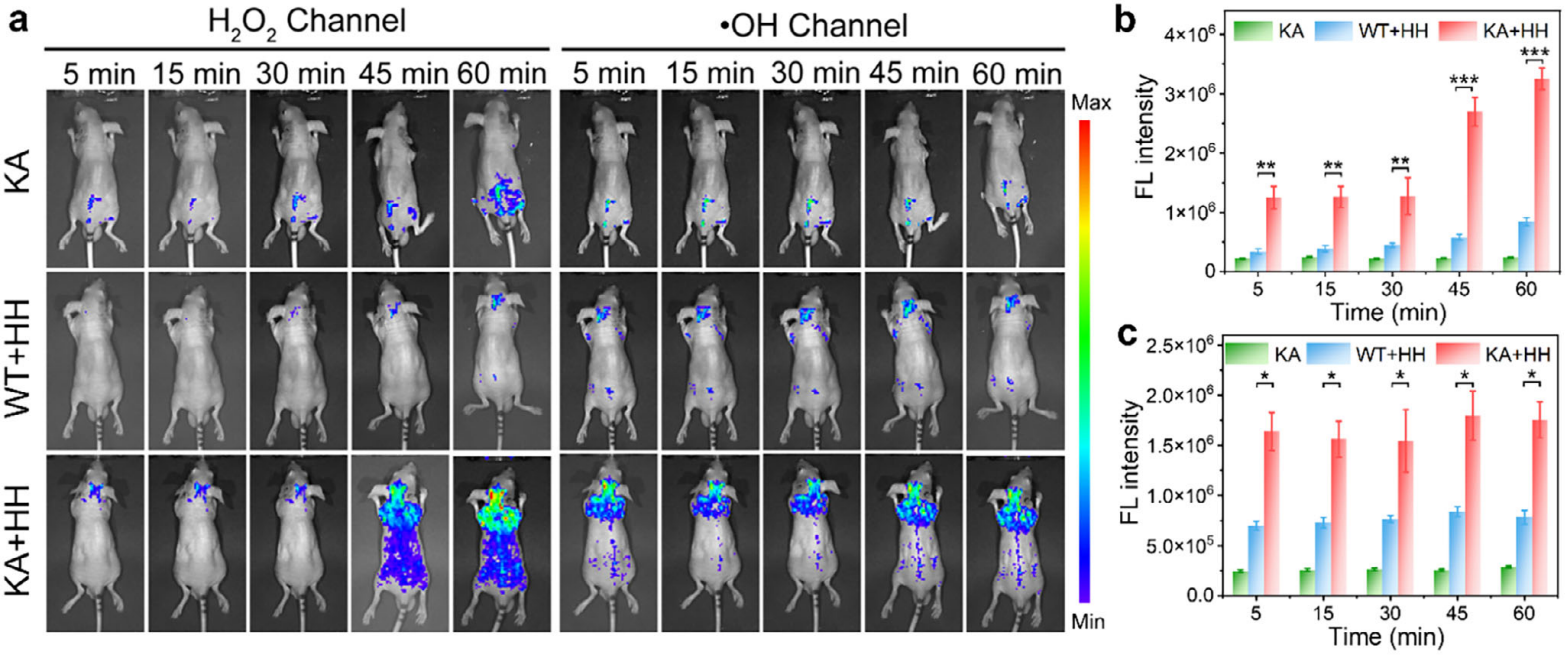
(a) Images of healthy and KA‐induced epileptic mice at 5, 15, 30, 45, 60 min after intravenous injection (i.v.) of Probe **HH**. Epileptic mice induced by intraperitoneal injection of KA (5 mg kg^−1^) for 12 h. The probe was injected into the tail vein at a dose of 0.25 mg/kg. Using the vivo imaging system (IVIS) spectrum imaging system. (b,c) Corresponding time‐dependent quantification of fluorescence (FL) intensity in the H_2_O_2_ (b) and •OH (c) channels. Significant differences (^⁎^ *p* < 0.05; ^⁎⁎^ *p* <0.01; ^⁎⁎⁎^ *p* < 0.001) are performed by two‐tailed Student's t‐test.

To further validate the probe's in vivo performance in a mammalian system, we tracked the dynamic fluorescence changes in KA‐induced epileptic mice. Prior to conducting the in vivo fluorescence experiments, the KA‐induced epilepsy model in mice was confirmed through Nissl staining. Relative to the control slices, in which pyramidal neurons were tightly packed and intensely stained, the KA group showed a marked reduction in neuronal number, shrunken cell bodies with pale cytoplasmic staining, and condensed nuclei (Figure S23), consistent with previous reports [56, 57], Nissl staining revealed characteristic neuronal injury in KA‐treated mice, supporting successful establishment of the epilepsy model. Probe **HH** was intravenously injected into both healthy and epileptic mice, and real‐time fluorescence imaging was performed at several post‐injection intervals. As shown in Figure 5, almost no fluorescence signal was detected in the brains of KA model control mice that did not receive the probe. After probe injection, the fluorescence signal in the brains of normal mice gradually increased over time, with the green channel (•OH) showing a faster rise. In contrast, the brains of KA‑induced epileptic mice exhibited not only a more rapid fluorescence onset but also significantly higher fluorescence intensity. To more precisely compare the trends of ROS levels in the brains of epileptic and normal mice, we further injected probe **HH** precisely into the mouse hippocampus using a stereotaxic apparatus (Figure S26). The results showed that the fluorescence signals in both channels were also significantly higher in the brains of KA‑induced epileptic mice than in normal mice, fully demonstrating that H_2_O_2_ and •OH levels are markedly elevated in the brain during epilepsy. After completing the in vivo imaging, we collected the brains and major organs of the mice for ex vivo imaging and histological analysis to determine the fluorescence distribution of the probe, the true origin of the signals, and the biosafety of the probe. The ex vivo imaging results of the brain and major organs (Figure S25) showed that the probe possessed a certain degree of blood–brain barrier permeability, and the ex vivo fluorescence signals were consistent with those observed in vivo, confirming the authenticity and reliability of the brain fluorescence signals. Furthermore, H&E staining of the heart, liver, spleen, lungs, and kidneys indicated that neither healthy mice nor KA‑induced epileptic model mice showed any tissue damage or inflammatory lesions after administration of the probe **HH**, demonstrating that the probe has good biocompatibility (Figure S24).

These in vivo findings are consistent with our cellular observations and confirm the capability of probe **HH** to sensitively and selectively detect dynamic ROS changes across both chronic and acute epilepsy models. Furthermore, the distinct temporal profiles of H_2_O_2_ and •OH may provide useful information for understanding oxidative stress‐associated changes during seizure‐like processes. Altogether, probe HH demonstrates robust in vivo imaging performance and holds considerable promise as a tool for monitoring ROS dynamics and evaluating oxidative stress‐related therapeutic responses in epilepsy and other redox‐associated neurological disorders.

## 3 Conclusion

We have developed a dual‐responsive fluorescent probe, **HH**, for the simultaneous and selective detection of H_2_O_2_ and •OH in complex biological systems. This molecular design leverages a unique aromatic hydroxylation mechanism for •OH and a boronate ester moiety for H_2_O_2_ recognition and, combined with position‐specific cyclization of cyanide groups to yield two distinct fluorescent outputs. The resulting probe exhibits excellent optical properties, high selectivity and sensitivity, and low cytotoxicity, enabling reliable real‐time imaging of ROS in live cells and in vivo models. Owing to this architecture, **HH** displays favorable photophysical properties, excellent selectivity and sensitivity, and minimal cytotoxicity, enabling robust real‐time monitoring of ROS in living cells and in vivo systems.

Probe **HH** has been successfully applied to monitor dynamic changes in H_2_O_2_ and •OH during gasdermin‐mediated pyroptosis, revealing a strong correlation between ROS accumulation and pyroptotic severity. In addition, **HH** enabled dual‐channel imaging of H_2_O_2_ and •OH in seizure‐like cellular and animal models, including PTZ‐induced zebrafish and KA‐induced mouse models. To the best of our knowledge, few fluorescent probes have been reported for dual‐channel imaging of H_2_O_2_ and •OH in epilepsy‐related models. These findings support the involvement of oxidative stress in epilepsy‐related pathology and indicate that **HH** is a useful tool for probing redox‐associated molecular processes. Moreover, **HH** shows potential utility in evaluating the antioxidative efficacy of candidate antiepileptic drugs, suggesting its suitability for therapeutic screening applications. Altogether, this work not only offers a versatile imaging strategy for probing ROS dynamics in neurological disease but also opens new avenues for investigating the broader roles of H_2_O_2_ and •OH in inflammation, neurodegeneration, and therapeutic intervention.

## 4 Experimental Section

General Information and Methods: The chemicals were purchased from Aladdin (Shanghai, China) and used without further purification. Solvents used were purified by standard methods prior to use. Ultrapure water (18.25 MΩ cm) was used throughout all experiments (Aquapro water ultrapurification system). All reactions were magnetically stirred and monitored by thin layer chromatography (TLC). Column chromatography was conducted over silica gel (200–300 mesh). UV–vis absorption spectra were collected on a UV‐2600i spectrophotometer (Shimadzu Co., Japan). Fluorescence spectra were recorded on Hitachi F‐7100 spectrophotometer (Hitachi Ltd, Japan) with a 1 cm standard quartz cell (PMT voltage, 700 V; Scan speed, 1200 nm/min; Delay, 0 s; Response, 2 s). The fluorescence images of solutions were excited by a 365 nm lighting of ZF‐20D UV analyzer (Shanghai, China). The visual pictures were captured by a smartphone built‐in camera. ^1^H NMR and ^13^C NMR spectra were obtained with tetramethyl silane (TMS) as the internal standard on a BRUKER AVANCE‐500 spectrometer and the chemical shifts (δ) were expressed in ppm and coupling constants (*J*) in Hertz. FT‐IR spectra were collected on a Nippon‐Shimadzu‐IR Tracer 100 Fourier transform infrared (FT‐IR) spectrophotometer (KBr bullet method). High resolution mass analyses were performed using Agilent 1290/6545 UHPLC‐QTOF/MS mass spectrometer system. The flow cytometry analyses were performed on Cytek DxP AthenaTM V2‐B4‐R2 flow cytometer (Cytek, China). All of the cell and zebrafish imaging experiments were conducted on fluorescence confocal microscope system (Leica SP8, Germany). The in vivo imaging experiments was performed with IVIS imaging system.

Synthesis of the Probe **HH**: To a solution of compound **4** (0.2 g, 657.15 µmol) in acetonitrile (15 mL) was added 2‐(4‐(bromomethyl)phenyl)‐4,4,5,5‐tetramethyl‐1,3,2‐dioxaborolane (0.585 g, 1.97 mmol). Then add K_2_CO_3_ (0.182 g, 1.31 mmol). The reaction mixture was stirred 95 °C overnight. After completion of the reaction, the reaction mixture was dried under vacuum then crude directly used in the next step without purification. The resulting residue was dissolved in ethanol (15 mL), to which malononitrile (0.023 g, 307 µmol) was added. The reaction was stirred at room temperature overnight. After completion of the reaction, the reaction mixture was dried under vacuum then crude was purified by silica gel chromatography to afford probe **HH** (45 mg, 12% yield) ^1^H NMR (500 MHz, CDCl_3_) δ 8.05 (s, 1H), 7.86 (d, *J* = 7.9 Hz, 2H), 7.44 (s, 1H), 7.40 (d, *J* = 7.8 Hz, 2H), 5.82 (s, 1H), 5.13 (s, 2H), 3.70 (s, 4H), 3.67–3.61 (m, 1H), 3.60–3.54 (m, 2H), 3.43 (t, *J* = 9.9 Hz, 1H), 3.28 (m, *J* = 10.2, 7.6 Hz, 1H), 2.82–2.74 (m, 2H), 2.63 (m, *J* = 16.6, 5.5 Hz, 1H), 2.16–2.15 (m, *J* = 11.2, 6.1, 5.6 Hz, 2H), 2.05–1.98 (m, 1H), 1.51–1.44 (m, 1H), 1.38 (s, 12H). ^13^C NMR (126 MHz, CDCl_3_) δ 172.98, 156.17, 149.74, 144.13, 139.16, 135.24, 127.35, 126.58, 117.78, 116.91, 109.58, 106.72, 93.40, 83.95, 70.89, 66.32, 57.78, 52.21, 51.75, 47.31, 47.23, 31.08, 29.89, 24.89, 23.17. IR (KBr pellet, cm^−1^):3446.06, 2976.05, 2203.63, 1734.18, 1612.03, 1563.62, 1541.05, 1511.26, 1488.09, 1406.97, 1357.57, 1388.86, 1298.90 (Scheme S2, All intermediates and final products were thoroughly characterized using ^1^H NMR, ^13^C NMR, IR spectroscopy, and high‐resolution mass spectrometry. Figures S27–S30 and S41–S43).

Cell Culture: HeLa and SH‐SY5Y cells were obtained from China Center for Type Culture Collection (Wuhan, China). The HeLa and SH‐SY5Y cells were maintained in Dulbecco's modified Eagle's medium (DMEM) with 10% (V/V) heat‐inactivated fetal bovine serum (FBS) (Gibco BRL, Grand Island, NY, USA), and 1% streptomycin‐penicillin (10 000 µg mL^−1^ and 10 000 U mL^−1^) in a humidified incubator of 5% CO_2_ and 95% air at 37°C.

Imaging of endogenous and exogenous •OH and H_2_O_2_ in living cells: For fluorescence imaging of endogenous and exogenous •OH and H_2_O_2_, HeLa and SH‐SY5Y cells were cultured in serum‐free DMEM containing 0.5% (v/v) DMSO. For endogenous ROS imaging, cells were incubated with probe **HH** (5 µm) for 30 min, washed three times with phosphate‐buffered saline (PBS), and directly subjected to confocal imaging. For exogenous ROS imaging, cells were first pretreated with 2,2,6,6‐tetramethyl‐1‐piperidinyloxy (TEMPO, 0.1 mm) or N‐acetyl‐L‐cysteine (NAC, 0.25 mm) for 30 min, followed by incubation with **HH** (5 µm) for an additional 30 min. Subsequently, exogenous •OH or H_2_O_2_ (100 µm) was added and incubated for another 30 min. After washing three times with PBS, the cells were subjected to fluorescence imaging. For monitoring endogenous ROS generation, cells were stimulated with lipopolysaccharide (LPS, 1 µg mL^−^^1^) and phorbol 12‐myristate 13‐acetate (PMA, 1 µg mL^−^^1^) for 30 min to induce intracellular ROS production. For ROS scavenging experiments, cells were pretreated with TEMPO (0.1 mm) or NAC (0.25 mm) for 30 min prior to incubation with **HH** (5 µm) for 30 min. After washing three times with PBS, fluorescence images were acquired. Cells were imaged by a fluorescence confocal microscope (Zeiss LSM 880, Germany). All experiments were repeated three times. (λ_ex_ = 458 nm, λ_em_ = 480 – 560 nm for the green channel; λ_ex_ = 476 nm, λ_em_ = 580–650 nm for the red channel).

### 4.1 Monitoring Intracellular •OH and H_2_O_2_ Change During Pyroptosis

HeLa cells were treated with 15/20 mm dimethyl‐α‐ketoglutarate (DM‐αKG) for appropriate time to induce pyroptosis. After that the culture media were removed, and the cells were washed with serum‐free media and then incubated with 5 µM probe for 30 min. To inhibit pyroptosis, the cells were incubated with 15 mm dimethyl‐α‐ketoglutarate in the presence of 50 µm Z‐VAD‐FMK, 100 µm TEMPO or 100 µm Catalase. Fluorescence imaging was performed with fluorescence confocal microscope. (*λ*_ex_ = 458 nm, *λ*_em_ = 480–560 nm for the green channel; *λ*_ex_ = 476 nm, *λ*_em_ = 580–650 nm for the red channel.

### 4.2 Visualization of •OH and H_2_O_2_ in SH‐SY5Y Cell Injury Model Stimulated by PTZ

SH‐SY5Y Cells were treated with 0.1, 0.3, and 0.5 mm pentylenetetrazole (PTZ) for 8 h to induce cell injury. After the cells were washed with PBS for three times and then incubated with 5 µm probe for 30 min. Fluorescence imaging was performed with fluorescence confocal microscope. (*λ*_ex_ = 458 nm, *λ*_em_ = 480–560 nm for the green channel; *λ*_ex_ = 476 nm, *λ*_em_ = 580–650 nm for the red channel).

### 4.3 Flow Cytometry Analysis

Flow cytometry assay was performed for the detection of •OH and H_2_O_2_ with probe **HH**. Cells were inoculated in six‐well plates of ≈2.0 × 10^5^ cells per well and then treated as described in cell imaging (Figure 4a). After that, cells were washed, digested by Trypsin‐EDTA Solution, and resuspended in PBS and analyzed by flow cytometry. Collection windows: Comp‐V5‐A: *λ*_ex_ = 405 nm, *λ*_em_ = 498–518 nm; Comp‐B6‐A: *λ*_ex_ = 488 nm, *λ*_em_ = 606–630 nm.

### 4.4 Imaging of Endogenous and Exogenous •OH and H_2_O_2_ in Zebrafish

We are grateful to Prof. Yun Deng (Zebrafish Genetics Laboratory, College of Life Science, Hunan Normal University, Changsha, China) for providing the normal zebrafish and epilepsy model zebrafish. All of the animal procedures were performed in accordance with the guidelines and approved by the Animal Ethics Committee (Hunan Normal University, No. 2022‐161). For the detection of endogenous •OH and H_2_O_2_ in living zebrafish, 8‐day‐old zebrafish were prepared. Zebrafish were incubated with probe **HH** (10 µm) in E3 water containing 0.5% DMSO (v/v) for 30 min, then imaged after washed by PBS for three times. To detect exogenous •OH and H_2_O_2_, zebrafish were pretreated with 2,2,6,6‐tetramethyl‐1‐piperidinyloxy (TEMPO, a scavenger of •OH, 0.1 mm) and N‐acetyl‐L‐cysteine (NAC, a scavenger of ROS, 0.25 mm) separately for 30 min, subsequently incubated with probe **HH** (10 µm, 30 min), then incubated with •OH and H_2_O_2_ (100 µm, 30 min), then washed with phosphate‐buffered saline (PBS) for three times. Zebrafish were imaged by a fluorescence confocal microscope (Zeiss LSM 880, Germany). All experiments were repeated three times. (*λ*_ex_ = 458 nm, *λ*_em_ = 480–560 nm for the green channel; *λ*_ex_ = 476 nm, *λ*_em_ = 580–650 nm for the red channel).

### 4.5 Imaging Epilepsy Model Zebrafish

We choose zebrafish with well‐developed nervous system at the 8‐day postfertilization (dpf) stage, incubated them with PTZ (6 mm) to induce epilepsy. The 8‐day‐old zebrafish were incubated with probe (10 µm) for 30 min, and then imaged after washing by PBS buffer, as the control group. To monitor the fluctuation of •OH and H_2_O_2_ in zebrafish under epilepsy, zebrafish were mediated by PTZ (6 mm) for 3,6,12 h, subsequently incubated with probe for 30 min, and then the zebrafish were imaged after washing by PBS buffer. Zebrafish were imaged by a fluorescence confocal microscope (Zeiss LSM 880, Germany). All experiments were repeated three times. (*λ*_ex_ = 458 nm, *λ*_em_ = 480–560 nm for the green channel; *λ*_ex_ = 476 nm, *λ*_em_ = 580–650 nm for the red channel).

### 4.6 Imaging of •OH and H_2_O_2_ in Normal Mice and Epilepsy Model Mice

Five‐week‐old female BALB/c nude mice (average body weight 18 ± 2 g, *n* = 6 mice per group) were purchased from Hunan SJA Laboratory Animal Co., Ltd. All animal experiments were approved by the Animal Ethics Committee of Hunan Normal University and performed in accordance with relevant guidelines (No. 2022‐161). Epilepsy was induced by intraperitoneal injection of kainic acid (KA, 5 mg kg^−1^), and the mice were observed for 12 h. Successful establishment of the epilepsy model was confirmed by a Racine score ≥ 4 or the appearance of typical epileptic behaviors (forelimb clonus, generalized tonic‐clonic seizures). The normal control group received an equal volume of saline. Fluorescence imaging was performed using an IVIS Spectrum imaging system (The Third Xiangya Hospital of Central South University) at 5, 15, 30, 45, and 60 min after tail vein injection of the probe (0.25 mg kg^−1^). (Red Channel: excitation filter, 465 nm; emission filter, 600 nm, Green Channel: excitation filter, 465 nm; emission filter, 520 nm). After imaging, the mice were euthanized, and major organs including the brain, heart, liver, spleen, lungs, and kidneys were collected for ex vivo fluorescence imaging. Significant differences (^⁎^ *p* < 0.05; ^⁎⁎^ *p* <0.01; ^⁎⁎⁎^ *p* < 0.001) are performed by two‐tailed Student's t‐test.

## Author Contributions

**Ting Yu**: methodology, validation, investigation. **Haoyu Jin**: methodology, investigation. **Peng Yin**: conceptualization, writing – review and editing, funding acquisition. **Youyu Zhang**: investigation. **Yuling Xu**: methodology, software, writing – original draft, investigation. **Yang Li**: methodology, formal analysis, investigation. **Xin Jiang**: methodology, software, investigation. **Haitao Li**: investigation. **Yabing Gan**: methodology, writing – original draft, software, data curation, investigation. **Jong Seung Kim**: conceptualization, writing – review and editing, funding acquisition.

## Conflicts of Interest

The authors declare no conflicts of interest.

## Supporting information




**Supporting File**: advs76679‐sup‐0001‐SuppMat.docx.

## Data Availability

The data that support the findings of this study are available from the corresponding author upon reasonable request.
