# Supplementary material for: Synchronized Imaging of Hydrogen Peroxide and Hydroxyl Radical in Pyroptosis and Epilepsy
Source: Adv Sci (Weinh). 2026 Jul 16;13(57):e76679. doi: 10.1002/advs.76679 (PMC13373897; doi:10.1002/advs.76679)
Supplement: Supplementary file 1 [file ADVS-13-e76679-s001.docx]

Supporting Information

Synchronized Imaging of Hydrogen Peroxide and Hydroxyl Radical in Pyroptosis and Epilepsy

Yabing Gan,^a#^ Yuling Xu,^b#^ Ting Yu,^a^ Yang Li,^a^ Xin Jiang,^a^ Haoyu Jin,^a^ Haitao Li,^a^ Youyu Zhang,^a^ Peng Yin,^a^* and Jong Seung Kim ^b,c^*

^a^ Institute of Interdisciplinary Studies, Key Laboratory of Chemical Biology and Traditional Chinese Medicine Research (Ministry of Education), College of Chemistry and Chemical Engineering, Hunan Normal University, Changsha, 410081, China.

^b^ Department of Chemistry, Korea University, Seoul 02841, Korea.

^c^ National Research Laboratory for Convergence Degradation Biology, Korea University, Seoul 02841, Korea.

Table of Contents

I. Experimental Section ………………………………………………………………………………S2

II. Supplementary Spectra and Figures…………………………….………….......………………….S12

III. ^1^H NMR and ^13^C NMR Spectra…………..............................................................................……S18

IV.References…….............................................……..............................……………………………S26

I. Experimental Section

General procedure for fluorescence and UV-visible measurements.

The stock solution of probe HH (10 mM) was prepared in DMF. Stock solutions (1×10^-2^ M) of •OH, H_2_O_2_, ClO^-^, ^1^O_2_, O_2_^•-^, TBHP, NO, ONOO^-^, Cys, Hcy, GSH, NAC, Na_2_SO_3_, Na_2_S, Al^3+^, Fe^2+^, Zn^2+^, K^+^, Ni^2+^, Cu^2+^, F^-^, I^-^, Cl^-^, Br^-^, Glu, Lys, Try, Gly, L-Thr and His were prepared in deionized water. These stock solutions were further diluted to required concentration for measurement. Test solutions were prepared as follows: 20 μL of probe HH solution (1.0 mM) and proper analyte’s solution were added into a test tube, and the solution was diluted to 2 mL using PBS buffer containing 20% EtOH (v/v). Absorption and fluorescence spectra were recorded in an indicated time at room temperature.

Generation of reactive oxygen species (ROS) and reactive nitrogen species (RNS).

Hydrogen peroxide (H_2_O_2_): Commercial hydrogen peroxide solution (30%) was diluted, and the concentration of H_2_O_2_ solution was determined by recording the UV/Vis absorption at 240 nm (ε = 43.6 M^-1^ cm^-1^); Sodium hypochlorite (NaOCl): After diluting commercial sodium hypochlorite solution, and the concentration of HOCl solution was determined by recording the UV/Vis absorption at 292 nm (ε = 350 M^-1^ cm^-1^); Tertbutylhydroperoxide (TBHP) were diluted from the commercially available solution to 0.1 M in ultrapure water; Hydroxyl radical (•OH): Hydroxyl radical solution (10 mM) was prepared by adding aqueous ferrous sulfate (11 mM) to aqueous hydrogen peroxide (10 mM) according to the Fenton reaction; Superoxide (O_2_^•-^):Superoxide was obtained by dissolving KO_2_ in dry DMSO, and the concentration of O_2_^•-^ solution was determined by recording the UV/Vis absorption at 250 nm (ε = 2690 M^-1^ cm^-1^); Singlet oxygen (^1^O_2_): Singlet oxygen was prepared by mixing HOCl (10 eq.) and H_2_O_2_ in the PBS buffer; Peroxynitrite (ONOO^-^): A mixed solution of sodium nitrite (0.6 M) and hydrogen peroxide (0.7 M) was acidified with hydrochloric acid solution (0.6 M) at 0 °C. Then NaOH (1.5 M) was added rapidly within 1-2 s to make the solution alkaline. The solution was passed through a short MnO_2_ column to remove excess H_2_O_2_.The concentration of the as-prepared ONOO^−^ was calibrated by measuring its absorbance at 302 nm (1670 M^-1^ cm^-1^) by UV-VIS absorption spectroscopy.

MTT assay for the cell cytotoxicity.

Cell cytotoxicity was evaluated by MTT assay. Cells were cultivated in a 96-well plate until 80–90% confluence, and incubated with different concentrations of probe HH (0-10 μM) for 24 h. Then 20 μL 3-(4,5-dimethyl-2-thiazolyl)-2,5-diphenyltetra zolium bromide (MTT, 5 mg/mL) was added for 4 h at 37 °C. After MTT was washed, 150 μL DMSO was added. Absorbance was measured at 570 nm with a multi-function microplate reader (SpectraMax i3, USA). All experiments were repeated six times, and the data were presented as the percentage of control cells.

Quantum yield measurements.

Fluorescein in 0.1 M NaOH aqueous solution (quantum yield is 0.95) were selected as references. The QY_s_ were determined by comparing the integrated fluorescence intensity and the absorbance value of the one sample with those of references. The absorbances less than 0.05 (highly diluted samples can minimize the second optical processes such as re-absorption and re-emission effects) at the excitation wavelength were recorded respectively. And the integrated fluorescence intensity was plotted against absorbance at the excitation wavelength and fitted into a linear function to obtain the slope. The two slopes (one obtained from the reference and the other from the sample) were used to calculate the QY_s_ using the equation:

QY_s_ = QY_r_(m_s_/m_r_)(n_s_^2^/n_r_^2^).

Where QY is the quantum yield, m is the slope determined by the curves. Besides, n is the refractive index (1.33 for 0.1 M NaOH solvent, 1.337 for PBS buffer at room temperature). The subscript “r” refers to the references and “s” refers to the samples. Based on the data measured in Figure S1 and the corresponding equations, the fluorescence quantum yields (Φ) of probe HH in response to H_2_O_2_ and •OH were calculated to be 0.05 and 0.09, respectively.

Calculation of the limit of detection (LOD)

LOD = 3σ*/S*

σ: the standard deviation of the blank solution.

𝑥̅ is the mean of the blank measures; 𝑥_𝑖_ is the values of blank measures; n is the number of tested blank measure (n = 10)

*S*: the slope of the linear calibration plot between the fluorescence emission intensity and the concentration of •OH and H_2_O_2_.

Statistical Analysis.
All data were repeated six times and are expressed as mean ± standard deviation (SD) unless otherwise stated. Means were compared using a two-sided Student’s t test, p < 0.1, p < 0.01, and p < 0.001 were considered to be statistically significant as shown by *, ** and ***, respectively. All data were analyzed by GraphPad Prism 8 and OriginLab 2021.

Fluorescence Quantification.

Fluorescence images were analyzed using the built-in ROI analysis module of the Leica SP8 software . All fluorescence images were acquired using identical microscope settings, including laser power, detector gain, pinhole size, scan speed, image resolution, and other acquisition parameters. For each experimental group, multiple randomly selected microscopic fields from three independent biological experiments were analyzed. From each microscopic field, 10–20 representative individual cells were randomly selected according to identical ROI selection criteria. The fluorescence intensity of each individual cell was quantified by measuring the Mean Value (mean fluorescence intensity) within the corresponding ROI. Fluorescence measurements from all analyzed cells were subsequently imported into GraphPad Prism 8 for statistical analysis. The green and red fluorescence channels were analyzed independently throughout the entire workflow. For each fluorescence channel, the measured fluorescence intensities were normalized to the corresponding control group, which was defined as 1.0, and the results are presented as relative fluorescence intensities. The average fluorescence intensity from all analyzed cells was used for statistical analysis.

Scheme S1. Strategies for detecting hydrogen peroxide and hydroxyl radicals.

Scheme S2. Synthesis of the probe HH.

Synthesis of the Compound 3.

Compound 2 was prepared according to our previous work[1]. Compound 2 (10.0 g, 10.33 mmol), acetic acid (6 mL), and hydrobromic acid (48%, 8 mL) were combined in a round-bottom flask. The mixture was stirred under an argon atmosphere at reflux for 6 hours. Upon reaction completion, the solvent was removed under reduced pressure. The crude compound 3 was unstable and directly used for the next step without further purification.

Synthesis of the Compound 4.

Under Argon, fresh distilled DMF (4 mL) was added dropwise to POCl_3_ (1.8 mL) at room temperature and stirred for 30 min to yield a red solution. Then directly spin dried compound 3 (2.86 g, 10.35 mmol, dissolved in 20 mL DMF) was added dropwise to the above solution and a scarlet suspension was yielded. The mixture was stirred at 80 °C for 6 h until the reaction was completed, then poured into 150 mL of ice water. NaOH solution (20%) was added to adjust the pH to 6. The mixture was extracted with ethyl acetate. Combined ethyl acetate extracts were washed with water, dried over anhydrous Na_2_SO_4_ and vacuum evaporated. The residue was purified by silica gel chromatography to afford compound 4 (1.2 g, 38.1%). ^1^H NMR (500 MHz, CDCl_3_) δ 11.68 (s, 1H), 9.43 (s, 1H), 6.46 (s, 1H), 5.85 (s, 1H), 3.69 (s, 3H), 3.65–3.59 (m, 2H), 3.57–3.51 (m, 1H), 3.50–3.40 (m, 2H), 3.28 (m, 1H), 2.27–2.58 (m, 2H), 2.57–2.55 (m, 1H), 2.53–2.13 (m, 2H), 2.12–1.97 (m, 1H), 1.44–1.40 (m, 1H). ^13^C NMR (126 MHz, CDCl_3_) δ 191.62, 172.70, 159.51, 143.99, 126.37, 112.55, 110.15, 96.15, 57.11, 51.84, 51.70, 47.55, 47.36, 30.79, 30.05, 23.27.

Synthesis of the probe HH.

To a solution of compound 4 (0.2 g, 657.15 μmol) in acetonitrile (15 mL) was added 2-(4-(bromomethyl)phenyl)-4,4,5,5-tetramethyl-1,3,2-dioxaborolane (0.585 g, 1.97 mmol). Then add K_2_CO_3_ (0.182 g, 1.31 mmol). The reaction mixture was stirred 95 °C overnight. After completion of the reaction, the reaction mixture was dried under vacuum then crude directly used in the next step without purification. The resulting residue was dissolved in ethanol (15 mL), to which malononitrile (0.023 g, 307 μmol) was added. The reaction was stirred at room temperature overnight. After completion of the reaction, the reaction mixture was dried under vacuum then crude was purified by silica gel chromatography to afford probe HH (45 mg, 12% yield) ^1^H NMR (500 MHz, CDCl_3_) δ 8.05 (s, 1H), 7.86 (d, *J* = 7.9 Hz, 2H), 7.44 (s, 1H), 7.40 (d, *J* = 7.8 Hz, 2H), 5.82 (s, 1H), 5.13 (s, 2H), 3.70 (s, 4H), 3.67 – 3.61 (m, 1H), 3.60 – 3.54 (m, 2H), 3.43 (t, *J* = 9.9 Hz, 1H), 3.28 (m, *J* = 10.2, 7.6 Hz, 1H), 2.82 – 2.74 (m, 2H), 2.63 (m, *J* = 16.6, 5.5 Hz, 1H), 2.16 – 2.15 (m, *J* = 11.2, 6.1, 5.6 Hz, 2H), 2.05 – 1.98 (m, 1H), 1.51 – 1.44 (m, 1H), 1.38 (s, 12H). ^13^C NMR (126 MHz,CDCl_3_) δ 172.98, 156.17, 149.74, 144.13, 139.16, 135.24, 127.35, 126.58, 117.78, 116.91, 109.58, 106.72, 93.40, 83.95, 70.89, 66.32, 57.78, 52.21, 51.75, 47.31, 47.23, 31.08, 29.89, 24.89, 23.17. IR (KBrpellet, cm^-1^):3446.06, 2976.05, 2203.63, 1734.18, 1612.03, 1563.62, 1541.05, 1511.26, 1488.09, 1406.97, 1357.57, 1388.86, 1298.90.

Scheme S3. Synthesis of the compound 2-6.

Scheme S4. Proposed responding mechanism of probe HH for sensing •OH/H_2_O_2_.

Synthesis of the compound 2-2.

To 3-fluoro-2-nitroanisole (10 g, 58.4 mmol) in 200 mL of acetonitrile was added proline methyl ester hydrochloride (11.61 g, 70.1 mmol) and triethylamine (17.7 g, 175.3 mmol). The mixture was then heated to reflux and stirred for 8 hours. After cooled to room temperature, the mixture was filtered and acetonitrile was removed under reduced pressure. The mixture was diluted with water and extracted with ethyl acetate. The organic layer was separated and washed with brine. After dried by anhydrous Na_2_SO_4_, ethyl acetate was removed under reduced pressure. The resulting residue was dissolved in methanol (500 mL), to which zinc powder (16.7 g, 255.6 mmol) and ammonium chloride (13.7 g, 255.6 mmol) was added. The reaction was stirred at room temperature overnight. The mixture solution was filtered, and concentrated in vacuo. The crude material was dissolved in ethyl acetate, and washed with water. The organic layer was separated and dried by anhydrous Na_2_SO_4_. Then ethyl acetate was removed under reduced pressure. The resulting residue was dissolved in THF (400 mL). NaBH_4_ (9.7 g, 255.6 mmol) and boron trifluoride-diethyl etherate (255.6 mmol) were added. The mixture solution was heated to reflux for 8 hours. After cooled to room temperature, the reaction solution was treated with aqueous NaOH (20%) until the pH of the solution was 12. Then, the mixture was extracted with ethyl acetate, and washed with brine. After dried by anhydrous Na_2_SO_4_, the organic solvent was evaporated in vacuo. The resulting residue was purified by column chromatography. The compound 2-2 as a yellow oily liquid (4.2 g) was obtained in 48.3% yield for three steps. ^1^H NMR (500 MHz, CDCl_3_) δ 6.7 (t, *J* = 8.1 Hz, 1H), 6.3 (dd, *J* = 8.2, 1.2 Hz, 1H), 6.2 (dd, *J* = 8.0, 1.1 Hz, 1H), 3.8 (s, 3H), 3.6 – 3.5 (m, 2H), 3.4 – 3.4 (m, 1H), 3.3 – 3.2 (m, 1H), 2.8 (t, *J* = 9.4 Hz, 1H), 2.1 – 1.9 (m, 3H), 1.5 – 1.4 (m, 1H). ^13^C NMR (126 MHz, CDCl_3_) δ 146.48, 135.48, 121.69, 118.15, 105.11, 99.68, 56.77, 55.66, 47.80, 45.51, 29.92, 23.65.

Synthesis of the compound 2-3.

To a solution of compound 2-2 (4 g, 19.6 mmol) in acetonitrile (50 mL) was added iodoethane (3.66 g, 23.5 mmol). The reaction mixture was stirred at room temperature overnight. After completion of the reaction, the mixture was filtered and washed with hexane. The product was dried under vacuum to give a white solid 2-3 (3.9 g, 85.7% yield). ^1^H NMR (500 MHz, CDCl_3_) δ 7.2 (t, *J* = 8.3 Hz, 1H), 6.3 (dd, *J* = 8.3, 1.0 Hz, 1H), 6.2 (dd, *J* = 8.5, 1.0 Hz, 1H), 4.0 (dd, *J* = 13.0, 3.5 Hz, 1H), 3.9 (s, 3H), 3.9 – 3.8 (m, 1H), 3.6 – 3.4 (m, 3H), 3.2 – 3.1 (m, 1H), 3.0 (t, *J* = 12.6 Hz, 1H), 2.4 – 2.3 (m, 1H), 2.2 – 2.1 (m, 1H), 2.1 – 2.0 (m, 1H), 1.6 – 1.5 (m, 4H). ^13^C NMR (126 MHz, CDCl_3_) δ 152.59, 139.70, 131.32, 109.07, 105.47, 99.57, 56.73, 53.79, 50.91, 49.96, 48.34, 30.74, 24.19, 10.28. ^13^C NMR (126 MHz, CDCl_3_) δ 172.98, 156.17, 149.74, 144.13, 139.16, 135.24, 127.35, 126.58, 117.78, 116.91, 109.58, 106.72, 93.40, 83.95, 70.89, 66.32, 57.78, 52.21, 51.75, 47.31, 47.23, 31.08, 29.89, 24.89, 23.17.

Synthesis of the compound 2-4.

To a solution of compound 2-3 (3.5 g, 15 mmol) in dichloromethane (50 mL) was added boron tribromide (7.5 g, 30 mmol). The reaction mixture was stirred at 60°C overnight. After completion of the reaction, the reaction mixture was dried under vacuum, and then crude was purified by silica gel chromatography to afford compound 2-4 (1.8 g, 54.7% yield). ^1^H NMR (500 MHz, CDCl_3_) δ 6.9 (td, *J* = 8.0, 1.9 Hz, 1H), 6.3 (d, *J* = 8.0 Hz, 1H), 6.1 (d, *J* = 8.2 Hz, 1H), 3.7 – 3.6 (m, 1H), 3.5 (t, *J* = 8.7 Hz, 1H), 3.4 (dd, *J* = 13.1, 3.8 Hz, 1H), 3.2 – 3.1 (m, 1H), 2.9 – 2.8 (m, 1H), 2.7 (dd, *J* = 13.2, 6.8 Hz, 1H), 2.2 (dd, *J* = 13.3, 10.8 Hz, 1H), 2.1 – 2.1 (m, 2H), 2.0 – 2.0 (m, 1H), 1.4 – 1.3 (m, 1H), 1.3 – 1.2 (m, 3H). ^13^C NMR (126 MHz, CDCl_3_) δ 150.52, 140.04, 125.25, 120.48, 103.18, 101.90, 50.44, 49.50, 48.37, 48.24, 30.56, 24.23, 13.95, 1.05.

Synthesis of the compound 2-5.

Under Argon, fresh distilled DMF (3 mL) was added dropwise to POCl_3_ (1.3 mL) at room temperature and stirred for 30 minutes to yield a red solution. Then a portion of compound 2-4 (1.5 g, 6.87 mmol, dissolved in 20 mL DMF) was added dropwise to the above solution and a scarlet suspension was yielded. The mixture was stirred at 70 ºC for 12 hours until the reaction was completed, then poured into 150 mL of ice water. NaOH solution (20 %) was added to adjust the pH to 6. The mixture was extracted with ethyl acetate. Combined ethyl acetate extracts were washed with water, dried over anhydrous Na_2_SO_4_ and vacuum evaporated. The residue was purified by silica gel chromatography to afford compound 2-5 (0.45 g, 26.6%). ^1^H NMR (500 MHz, CDCl_3_) δ 11.9 (s, 1H), 9.5 (d, *J* = 3.9 Hz, 1H), 7.0 (dd, *J* = 8.7, 3.8 Hz, 1H), 6.2 (dd, *J* = 8.7, 3.8 Hz, 1H), 3.7 – 3.5 (m, 1H), 3.5 – 3.3 (m, 3H), 3.2 – 3.1 (m, 1H), 2.8 – 2.7 (m, 1H), 2.3 – 2.2 (m, 1H), 2.2 – 2.1 (m, 2H), 2.0 – 1.9 (m, 1H), 1.5 – 1.4 (m, 1H), 1.3 – 1.2 (m, 3H). ^13^C NMR (126 MHz, CDCl_3_) δ 192.75, 155.80, 144.67, 129.55, 118.95, 111.86, 104.14, 51.70, 49.39, 49.26, 47.65, 30.08, 23.43, 13.86.

Synthesis of the compound 2-6.

To a solution of compound 2-5 (0.1 g, 405.9 μmol) in ethanol (8 mL) was added malononitrile (32.2 mg, 487.2μmol). The reaction mixture was stirred at room temperature overnight. After completion of the reaction, the mixture was filtered and washed with ethanol. The product was dried under vacuum to give a yellow solid 2-6 (50 mg, 41.8%).^1^H NMR (500 MHz, DMSO-*d*_6_) δ 8.4 (d, *J* = 9.1 Hz, 1H), 7.4 (s, 2H), 6.7 (d, *J* = 9.2 Hz, 1H), 3.6 (d, *J* = 9.3 Hz, 1H), 3.5 (dd, *J* = 13.5, 3.4 Hz, 1H), 3.3 – 3.2 (m, 1H), 3.1 – 3.0 (m, 1H), 2.9 – 2.8 (m, 1H), 2.2 – 1.9 (m, 4H), 1.5 – 1.4 (m, 1H), 1.2 (t, *J* = 7.0 Hz, 3H).

Synthesis of the proposed product from the reaction between probe HH with H_2_O_2_ (HH-2).

To a solution of probe HH (20.0 mg, 143.5 μmol) in 5 mL acetonitrile and then 0.2 μL 30 % H_2_O_2_ solution was added. The reaction mixture was stirred at room temperature for 2 h under Argon. After completion of the reaction, the reaction mixture was dried under vacuum, and then crude was purified by silica gel chromatography to afford compound HH-2 (5.0 mg, 40.0%) was obtained by column chromatography. ^1^H NMR (500 MHz, CDCl_3_ ) δ 7.49 (d, *J* = 3.9 Hz, 1H), 6.27 (d, *J* = 3.8 Hz, 1H), 6.06 (d, *J* = 4.8 Hz, 1H), 3.69 (m, 1H), 3.43 (m, 3H), 3.27 (m, 2H), 2.69 (t, *J* = 10.1 Hz, 1H), 2.15 (m, 2H), 2.04 (m, 1H), 1.47 (m, 1H), 1.16 (d, *J* = 6.4 Hz, 3H). ^13^C NMR (126 MHz, CDCl_3_) δ 158.54, 150.96, 145.52, 142.13, 130.61, 116.93, 107.29, 106.52, 96.17, 94.16, 57.27, 50.22, 47.61, 45.71, 30.33, 23.39, 10.13. IR (KBr pellet, cm^-1^): 3308, 2963, 2925, 2868, 2213, 1649, 1604, 1525, 1459, 1420, 1336, 1233, 1185, 1123, 1081.

Table S1. Fluorescent Probes for Detection of Epilepsy-Related Biomarkers.

| Probe structure | Biomarkers | Biological applications | Biological model | Ref. |
| --- | --- | --- | --- | --- |
|  | O_2_^•−^ | Cell/ Mice | Epilepsy | [2] |
|  | Lipid droplets (LDs) | Cell/ Mice | Epilepsy | [3] |
|  | O_3_ | Cell | Epilepsy | [4] |
|  | Cys | Cell/ Mice | Epilepsy | [5] |
|  | O_2_^•−^ | Cell/ Mice Tissue | Epilepsy | [6] |
|  | Zn^2+^ | Cell/ Tissue | Epilepsy | [7] |
|  | Cys/Hcy/ GSH/ATP | Cell/ Zebrafish Tissue | Epilepsy/ Liver Injury | [8] |
|  | Cys | Cell/ Zebrafish  Mice | Epilepsy | [9] |
|  | Norepinephrine | Cell/ Tissue | Epilepsy | [10] |
|  | O_2_^•−^ | Cell/ Mice | Epilepsy | [11] |
|  | O_2_^•−^ | Cell/ Zebrafish  Mice | Epilepsy | [12] |
|  | ONOO^-^/ LDs | Cell/ Mice | Epilepsy | [13] |
|  | Cys | Cell/ Zebrafish | Epilepsy/ Food analysis | [14] |
|  | ONOO^-^ | Cell/ Mice | Epilepsy | [15] |
|  | ONOO^-^/Viscosity | Cell/ Mice | Epilepsy/ | [16] |
|  | O_2_^•−^ | Cell/ Mice Tissue | Epilepsy/non-alcoholic fatty liver /tumoral  ferroptosis models | [17] |
|  | ONOO^-^ | Cell/ Mice | Epilepsy | [18] |
|  | ONOO^-^ | Cell/ Mice | Epilepsy | [19] |
|  | Formaldehyde | Cell/ Mice Tissue | Epilepsy | [20] |
|  | Acetylcholinesteras | Cell/ Zebrafish  Mice | Epilepsy | [21] |
|  | Fe^2+^ | Tissue | Epilepsy | [22] |
|  | Thiol | Cell/ Mice | Epilepsy | [23] |
|  | HOCl | Cell/ Mice | Epilepsy | [24] |
|  | H_2_O_2_/•OH | Cell/ Zebrafish/  Mice | Epilepsy/ Pyroptosis | This work |

Table S2. Fluorescent probes for detecting two types of ROS.

| Probe structure | Biomarkers | *λ*_ex_ / *λ*_em_ | Ref. |
| --- | --- | --- | --- |
|  | HOCl/•OH | *λ*_ex= 410 nm_ / *λ*_em = 490 nm_ for •OH_；_ *λ*_ex= 490 nm_ / *λ*_em = 520 nm_ for HOCl | [25] |
|  | H_2_O_2_/HOCl | *λ*_ex= 400 nm_ / *λ*_em = 501 nm_ for HOCl_；_ *λ*_ex= 470 nm_ / *λ*_em = 685 nm_ for H_2_O_2_ | [26] |
|  | H_2_O_2_/HOCl | *λ*_ex= 350 nm_ / *λ*_em = 504 nm_ for HOCl_；_ *λ*_ex= 430 nm_ / *λ*_em =640 nm_ for H_2_O_2._ | [27] |
|  | H_2_O_2_/HOCl | *λ*_ex= 376 nm_ / *λ*_em = 409 nm_ for H_2_O_2；_ *λ*_ex= 440 nm_ / I_520_ /I_640 nm_ for HOCl_._ | [28] |
|  | H_2_O_2_/ONOO^-^ | *λ*_ex= 660 nm_ / *λ*_em = 704 nm_ for H_2_O_2；_ *λ*_ex= 360 nm_ / *λ*_em = 460 nm_ for ONOO^-^ | [29] |
|  | O_2_^•-^/ONOO^-^ | *λ*_ex= 675 nm_ / *λ*_em = 710 nm_ for H_2_O_2；_ *λ*_ex= 720 nm_ / *λ*_em = 466 nm_ for ONOO^-^ | [30] |
|  | HOCl/ONOO^-^ | *λ*_ex= 440 nm_ / I_520_ /I_640 nm_ for HOCl_；_ *λ*_ex= 400 nm_ / I_450_ /I_640 nm_ for ONOO^-^ | [31] |
|  | HOCl/ONOO^-^ | *λ*_ex= 595 nm_ / *λ*_em = 700 nm_ for HOCl_；_ *λ*_ex= 500 nm_ / *λ*_em = 570 nm_ for ONOO^-^ sequence-activatable | [32] |
|  | HOCl/ONOO^-^ | *λ*_ex= 631 nm_ / *λ*_em = 669 nm_ for ONOO^-^_；_ *λ*_ex= 407 nm_ / *λ*_em = 468 nm_ for HOCl | [33] |
|  | O_2_^•-^/ONOO^-^ | *λ*_ex= 595 nm_ / *λ*_em = 700 nm_ for O_2_^•-^_；_ *λ*_ex= 500 nm_ / *λ*_em = 570 nm_ for ONOO^-^ sequence-activatable | [34] |
|  | HOCl/ONOO^-^ | HOCl+ONOO^-^ sequence-activatable *λ*_ex= 450 nm_ / *λ*_em = 550 nm_ | [35] |
|  | HOCl/ONOO^-^ | *λ*_ex= 400 nm_ / *λ*_em = 464 nm_ for HOCl_；_ *λ*_ex= 540 nm_ / *λ*_em = 577 nm_ for ONOO^-^ | [36] |
|  | HOCl/ONOO^-^ | *λ*_ex= 518 nm_ / *λ*_em = 574 nm_ for HOCl_；_ *λ*_ex= 420 nm_ / *λ*_em = 520 nm_ for ONOO^-^ | [37] |
|  | H_2_O_2_/•OH | *λ*_ex= 440 nm_ / *λ*_em = 492 nm_ for •OH_；_ *λ*_ex= 472 nm_ / *λ*_em = 598 nm_ for H_2_O_2_ | This work |

II. Results and Discussion

## Supplementary Spectra and Figures

Figure. S1. Calibration curves for the determination of fluorescence quantum yields of rhodamine 6G, probe + H_2_O_2_, and probe + •OH. Using fluorescein as a reference (in 0.1 M NaOH aqueous solution, quantum yield Φ = 0.95), the relative fluorescence quantum yields were calculated by plotting the integrated fluorescence intensity against absorbance and comparing the slopes of the samples with that of the reference.

Figure. S2. (a) Fluorescence intensity spectra of probe (10 μM) in the presence of 100 μM H_2_O_2_ upon addition of 50 μM to 90 μM •OH in EtOH/PBS buffer (v/v = 2:8) with *λ*_ex_=440 nm. (b) Fluorescence intensity spectra of probe (10 μM) in the presence of 15 μM •OH upon addition of 50 μM to 90 μM H_2_O_2_ in EtOH/PBS buffer (v/v = 2:8) (*λ*_ex_=440 nm).

Figure. S3. (a) Fluorescence emission spectra of probe HH (10 μM) in the absence and presence of H_2_O_2_, and H_2_O_2_ + •OH in EtOH/PBS (10 mM, pH = 7.4, v/v, 2/8) at room temperature. *λ*_ex_ = 472 nm. Probe only (black line); (b) Probe + 30 equiv. H_2_O_2_ (red line); (c) Probe after complete reaction with 30 equiv. H₂O₂, followed by addition of 10 equiv. •OH (light red line); (b) Fluorescence emission spectra of probe HH (10 μM) in the absence and presence of •OH, and •OH + H_2_O_2_ in EtOH/PBS (10 mM, pH = 7.4, v/v, 2/8) at room temperature. *λ*_ex_ = 472 nm. Probe only (black line); (b) Probe + 10 equiv. •OH (green line); (c) Probe after complete reaction with 10 equiv. •OH, followed by addition of 30 equiv. H_2_O_2_ (light green line)

Figure. S4. (a) Fluorescence spectra of probe HH (10 μM) upon addition of •OH and various competing species in EtOH/PBS buffer (v/v = 2:8) with *λ*_ex_ = 440 nm. (b) Corresponding fluorescence intensities at 492 nm for different analytes (1, blank; 2, ClO⁻; 3, ¹O₂; 4, O₂^•⁻^; 5, TBHP; 6, NO; 7, ONOO⁻; 8, Cys; 9, Hcy; 10, GSH; 11, NAC; 12, Na₂SO₃; 13, Na₂S; 14, Al³⁺; 15, Fe²⁺; 16, Zn²⁺; 17, K⁺; 18, Ni²⁺; 19, Cu²⁺; 20, F⁻; 21, I⁻; 22, Cl⁻; 23, Br⁻; 24, Glu; 25, Lys; 26, Try; 27, Gly; 28, L-Thr; 29, His; 30, •OH; 100 μM each).

Figure. S5. (a) Fluorescence spectra of probe HH (10 μM) upon addition of H_2_O_2_ and various competing species in EtOH/PBS buffer (v/v = 2:8) with *λ*_ex_ = 472 nm. (b) Corresponding fluorescence intensities at 598 nm for different analytes (1, blank; 2, ClO⁻; 3, ^1^O_2_; 4, O_2_^•⁻;^ 5, TBHP; 6, NO; 7, ONOO⁻; 8, Cys; 9, Hcy; 10, GSH; 11, NAC; 12, Na_2_SO_3_; 13, Na_2_S; 14, Al^3+^; 15, Fe^2+^; 16, Zn^2+^; 17, K^+^; 18, Ni^2+^; 19, Cu^2+^; 20, F^-^; 21, I⁻; 22, Cl⁻; 23, Br⁻; 24, Glu; 25, Lys; 26, Try; 27, Gly; 28, L-Thr; 29, His; 30, H_2_O_2_; 100 μM each).

 Figure. S6. (a) Time-dependent normalized fluorescence intensity changes at 598 nm for probe (10 μM) upon addition of 10 equiv. H_2_O_2_ for 30 min in EtOH/PBS buffer (v/v = 2:8) with *λ*_ex_ = 472 nm. (b) Time-dependent normalized fluorescence intensity changes at 492 nm for probe (10 μM) upon addition of 10 equiv. •OH for 30 min in EtOH/PBS buffer (v/v = 2:8) with *λ*_ex_ = 440 nm.

 Figure. S7. (a) and (b) Line plots of fluorescence intensity at 598 nm or 492 nm for probes (10 μM) in the absence and presence of H_2_O_2_ and •OH in EtOH/PBS buffer (v/v = 2:8) for 30 min at various pH (pH=3、4、5、6、7、8、9、10 and 11). *λ*_ex_ = 472 nm for H_2_O_2_, *λ*_ex_ = 440 nm for •OH, slit (nm): 5.0/5.0.

Figure. S8. (a) Absorption spectra (b) normalized fluorescence spectra of probe HH upon addition of •OH (10 equiv.) for 15 min (red) and the compound 2-6 (black) in EtOH/PBS buffer (v/v = 2:8) at room temperature. (c) The structure of HH-1 and compound 2-6.

Figure. S9. (a) Absorption spectra and (b) normalized fluorescence spectra of probe (Black) and probe upon addition of H_2_O_2_ (10 equiv.) for 15 min (blue) and the isolated Compound HH-2 (10 µM) from the reaction between probe with H_2_O_2_ (Red) in EtOH/PBS buffer (v/v = 2:8) at room temperature.

Figure. S10. HRMS spectrum of the reaction mixture of probe HH and •OH.

Figure. S11. HRMS spectrum of the reaction mixture of probe HH and H_2_O_2_.

Figure. S12. MTT assay for the survival rate of living SH-SY5Y and HeLa cells treated with various concentrations of probe for 24 h.

Figure. S13. (a) Confocal fluorescence imaging of endogenous and exogenous •OH and H_2_O_2_ in SH-SY5Y cells. (1A-3A) Cells were incubated with probe HH (5 µM) for 30 min. In the other groups, the cells were pretreated with LPS/PMA (1 μg·mL⁻¹) (1F-3F) /Tempo (0.1 mM) (1C-3C) and NAC (0.25 mM) (1B-3B) for 30 min, and then incubated with probe HH (5 μM, 30 min); Cells were pretreated with Tempo (0.1 mM)/ NAC (0.25 mM) for 30 min, subsequently incubated with probe HH (5 μM, 30 min), and finally incubated with •OH (100 μM) (1E-3E) / H_2_O_2_ (100 μM) (1D-3D) for 30 min. (*λ*_ex_ = 458 nm, *λ*_em_ = 480 - 560 nm for the green channel; *λ*_ex_ = 476 nm, *λ*_em_ = 580 - 650 nm for the red channel). Scale bar: 25 μm. (b) Relative pixel intensity of the fluorescence images 1A-3F in (a).

Figure. S14. (a) Confocal fluorescence images of endogenous and exogenous H_2_O_2_/·OH in zebrafishes. b) Quantification of the relative mean fluorescence levels of zebrafishes from the images of (a). (Green channel for •OH: *λ*_ex_ = 458 nm, *λ*_em_ = 480-560 nm; red channel for H_2_O_2_: *λ*_ex_ = 476 nm, *λ*_em_ = 580-650 nm. Scale bar: 250 μm). Scale bar: 0.25 mm. (b) Relative pixel intensity of the fluorescence images 1A-3E in (a).

Figure. S15. Confocal fluorescence imaging demonstrating the selectivity of probe HH toward ROS in SH-SY5Y cells. Cells were pretreated with NAC (0.2 mM, 30 min) and then incubated with probe HH (5 μM, 30 min), followed by treatment with various ROS ( 30 min): •OH (C1–C3,50 μM), H_2_O_2_ (D1–D3, 50 μM), HOCl (E1–E3, 50 μM), ^1^O_2_ (F1–F3, 50 μM), O_2_^•⁻^ (G1–G3,50 μM), or ONOO⁻ (H1–H3, 50 μM). Control (A1–A3) and NAC-only (B1–B3) groups were included for comparison. Green channel (•OH, *λ*ₑₓ = 458 nm, *λ*ₑₘ = 480-560 nm); Red channel (H_2_O_2_, *λ*ₑₓ = 476 nm, *λ*ₑₘ = 580–650 nm); Scale bar: 10 μm.

 Figure. S16. (a) Confocal fluorescence imaging for verifying the specificity of probe HH towards •OH in SH-SY5Y cells. Cells were incubated with probe HH (5 μM) for 30 min (Control group). For other groups, cells treated with exogenous •OH (50 μM) for 30 min (B1-D3), then pretreated with •OH scavengers DMSO (1%, C1-C3) or mannitol (50 mM,D1-D3) for 30 min, finally incubated with probe HH (5 μM) for another 30 min.·OH channel (green): *λ*ₑₓ = 458 nm, *λ*ₑₘ = 480-560 nm; H_2_O_2_ channel (red): *λ*ₑₓ = 476 nm, *λ*ₑₘ = 580–650 nm). Scale bar: 10 μm. (b) Relative pixel intensity of the fluorescence images A1-D3 in (a).

Figure. S17. Confocal fluorescence imaging for verifying the specificity of probe HH towards •OH in SH-SY5Y cells. Cells were treated as follows: (A1-A3) 5 μM probe for 30 min only; (B1-B3) cells treated with 100 μM CA and 5 μM probe; (C1-C3) cells pretreated with 5 mM mannitol (a ·OH scavenger) for 30 min after CA treatment (100 μM, 30 min), then treated with 5 μM probe for 30 min; (D1-D3) cells treated with 5 mM mannitol and 5 μM probe for 30 min, respectively. ·OH channel (green): *λ*ₑₓ = 458 nm, *λ*ₑₘ = 480-560 nm; H_2_O_2_ channel (red): *λ*ₑₓ = 476 nm, *λ*ₑₘ = 580–650 nm. Scale bar: 10 μm. (b) Relative pixel intensity of the fluorescence images A1-D3 in (a).

Figure. S18. Confocal fluorescence imaging to verify the elimination of H_2_O_2_ interference in the ·OH detection system in SH-SY5Y cells. Cells were incubated with probe HH (5 μM) for 30 min (A1-A3). For other groups, cells were pretreated with ·OH (50 μM, Excess Fe^2+^ to eliminate H_2_O_2_), and then incubated with probe HH (5 μM) for another 30 min(B1-B3). Cells were pretreated with H_2_O_2_ (50 μM), and then incubated with probe HH (5 μM) for another 30 min(C1-C3). Cells were pretreated with ·OH (50 μM) for 30 min, then incubated with catalase (CAT, 100 μM) for 30 min, and finally treated with probe HH (5 μM) for 30 min. ·OH channel (green): *λ*ₑₓ = 458 nm, *λ*ₑₘ = 480-560 nm; H_2_O_2_ channel (red): *λ*ₑₓ = 476 nm, *λ*ₑₘ = 580–650 nm. Scale bar: 10 μm. (b) Relative pixel intensity of the fluorescence images A1-D3 in (a).

Figure. S19. Effects of dimethyl-α-ketoglutarate on the viability of HeLa cells at different concentration in the absence and presence of Z-VAD-FMK (50 μM).

Figure. S20. Effect of DM-αKG at different concentrations on the morphology of HeLa cells incubated for 24 hours.

Figure. S21. (a) Monitoring of H_2_O_2_ and •OH generation in pyroptosis induced by DM-αKG. (A1-D3) Cells pretreated with 20 mM DM-αKG for 0 h, 4 h, 6 h or 8 h, and then incubated with 5 μM probe HH for 30 min. (E1-G3) Cells pretreated with 20 mM DM-αKG in the presence of (E1-E3) 50 μM Z-VAD-FMK, (F1-F3) 100 μM CAT or (G1-G3) 100 μM Tempo for 8 h, and then incubated with 5 μM probe HH for 30 min. Green channel for •OH: *λ*_ex_ = 458 nm, *λ*_em_ = 480-560 nm; red channel for H_2_O_2_: *λ*_ex_ = 476 nm, *λ*_em_ = 580-650 nm. Scale bar: 25 μm. (b) Relative pixel intensity of the fluorescence images A1-G3 in (a).

Figure.S22. (a) Fluorescence imaging •OH and H_2_O_2_ fluctuation in zebrafish by probe HH (10 μM). Zebrafish were incubated with probe (10 µM) for 30 min (A1-A3). In the other groups, the zebrafishes were pretreated with PTZ (6 mM) for 3, 6, 12 h (B1-D3); and finally incubated with probe (10 µM) for 30 min. (*λ*_ex_ = 458 nm, *λ*_em_ = 480 - 560 nm for the •OH channel; *λ*_ex_ = 476 nm, *λ*_em_ = 580 - 650 nm for the H_2_O_2_ channel). Scale bar: 0.5 mm. (b) Relative pixel intensity of fluorescence image of zebrafish brain in (a).

Figure. S23. Representative Nissl staining images of partial hippocampal sections from mice treated with PBS or kainic acid (KA, 5 mg kg⁻¹) for 12 h, showing the characteristic neuronal injury associated with successful establishment of the KA-induced epilepsy mode.

**Figure. S24.** Histological studies of mouse organ tissues treated with probe **HH** (0.25 mg/kg) and PBS for 24 h and H&E staining solution.

 **Figure. S25.** Images of healthy and KA-induced epileptic mice at 30 min after intravenous injection (i.v.) of Probe **HH**. Epileptic mice induced by intraperitoneal injection of KA (5 mg/kg) for 12 hours. The probe was injected into the tail vein at a dose of 0.25 mg/kg. Using the vivo imaging system (IVIS) spectrum imaging system. (b) Corresponding quantification of fluorescence intensity in the Fig. (a). Significant differences (^⁎^p < 0.05; ^⁎⁎^p <0.01; ^⁎⁎⁎^p < 0.001) are performed by two-tailed Student’s t-test.

**Figure. S26.** Images of healthy WT and epilepsy mice at 5, 15, 30, 45, 60 min. Epileptic mice induced by intraperitoneal injection of KA (5 mg/kg) for 12 hours. The probe **HH** was injected into the hippocampus of mice at a dose of 50 ng/μL (1 μL) using a stereotaxic device. Using the vivo imaging system (IVIS) Spectrum imaging system. (b) Corresponding quantification of fluorescence intensity in the Fig. (a). Red Channel: excitation filter, 465 nm; emission filter, 600 nm, Green Channel: excitation filter, 465 nm; emission filter, 520 nm. Significant differences (^⁎^*p* < 0.05; ^⁎⁎^*p* <0.01; ^⁎⁎⁎^*p* < 0.001) are performed by two-tailed Student’s t-test.

III. ^1^H NMR and ^13^C NMR Spectra

 Figure. S27. ^1^H NMR spectrum of compound 4 in CDCl_3_.

Figure. S28. ^13^C NMR spectrum of compound 4 in CDCl_3_.

Figure. S29. ^1^H NMR spectrum of the probe HH in CDCl_3_.

Figure. S30. ^13^C NMR spectrum of the probe HH in CDCl_3_.

Figure. S31. ^1^H NMR spectrum of compound 2-2 in DMSO-*d_6_*.

Figure.S32. ^13^C NMR spectrum of compound 2-2 in DMSO-*d_6_*.

Figure. S33. ^1^H NMR spectrum of compound 2-3 in CDCl_3_.

Figure. S34. ^13^C NMR spectrum of compound 2-3 in CDCl_3_.

Figure. S35. ^1^H NMR spectrum of compound 2-4 in CDCl_3_.

Figure. S36. ^13^H NMR spectrum of compound 2-4 in CDCl_3_.

Figure. S37. ^1^H NMR spectrum of compound 2-5 in DMSO-d*_6_*.

Figure. S38. ^13^C NMR spectrum of compound 2-5 in CDCl_3_.

Figure. S39. ^1^H NMR spectrum of compound 2-6 in DMSO-d*_6_*.

Figure. S40. ^1^H NMR spectrum of compound HH-2 in CDCl_3._

Figure. S41. IR spectrum of the probe HH.

Figure. S42. IR spectrum of the probe HH-2.

Figure. S43. High resolution mass spectrometry of probe HH.

IV. References

[1]	Y. Gan, G. Yin, X. Zhang, L. Zhou, Y. Zhang, H. Li, P. Yin, *Talanta* 2021, *225*, 122030.

[2]	W. Ying, F. Dong, Y. Shi, Z. Zhan, S. Wang, L. Lv, H. Liu, L. Liu, Y. Zheng, L. Zhang, *Dyes and Pigments* 2023, *213*, 111155.

[3]	J. Liu, M. Luo, W. Gao, K. Duan, H. Bian, Z. Jin, Y. Pan, S. Wang, Y. Gu, J. Zheng, R. Li, Z. Yuan, *Biosensors and Bioelectronics* 2025, *267*, 116774.

[4]	J. Zhang, Y. Duan, C. Liu, M. Zhang, H. Liu, X. Ming, Y. Li, X. Jiao, X. Wang, B. Tang, *Chemical Communications* 2025, *61* (44), 8043.

[5]	S. Li, P. Wang, M. Ye, K. Yang, D. Cheng, Z. Mao, L. He, Z. Liu, *Analytical Chemistry* 2023, *95* (11), 5133.

[6]	X. Luo, Z. Cheng, R. Wang, F. Yu, *Analytical Chemistry* 2021, *93* (4), 2490.

[7]	H. Santhakumar, R. V. Nair, D. S. Philips, S. J. Shenoy, A. Thekkuveettil, A. Ajayaghosh, R. S. Jayasree, *Scientific Reports* 2018, *8* (1), 9069.

[8]	T. Yu, Y. Li, J. Li, Y. Gan, Z. Long, Y. Deng, Y. Zhang, H. Li, P. Yin, S. Yao, *Advanced Science* 2025, *12* (11), 2415882.

[9]	S. Li, D. Song, W. Huang, Z. Li, Z. Liu, *Analytical Chemistry* 2020, *92* (3), 2802.

[10]	H. Yan, Y. Wang, F. Huo, C. Yin, *Journal of the American Chemical Society* 2023, *145* (5), 3229.

[11]	W. Yang, R. Liu, X. Yin, K. Wu, Z. Yan, X. Wang, G. Fan, Z. Tang, Y. Li, H. Jiang, *Analytical Chemistry* 2023, *95* (33), 12240.

[12]	J. Zhan, H. Huang, W. Quan, W. Lin, *Sensors and Actuators B: Chemical* 2025, *440*, 137887.

[13]	Y. Guo, H. Huang, Q. Zhang, H. Wang, M. Liu, W. Lin, *Analytica Chimica Acta* 2025, *1350*, 343863.

[14]	Y. Su, L. Li, P. Xiang, N. Liu, J. Huang, H. Zhou, Y. Deng, C. Peng, Z. Cao, Y. Fang, *Spectrochimica Acta Part A: Molecular and Biomolecular Spectroscopy* 2025, *324*, 124975.

[15]	G. Lu, H. Fan, K. Wang, G. Tian, C. Chen, Y. Wang, L. Wang, X. Fan, *Talanta* 2024, *267*, 125157.

[16]	Q. Zan, L. Fan, R. Wang, H. Wang, Y. Huang, X. Yu, Y. Zhang, C. Dong, S. Shuang, *Biosensors and Bioelectronics* 2025, *282*, 117495.

[17]	M. Si, L. Lv, Y. Shi, Z. Li, W. Zhai, X. Luo, L. Zhang, Y. Qian, *Analytical Chemistry* 2024, *96* (11), 4632.

[18]	L. Shen, H. Liu, M. Jin, J. Zhang, C. Yin, S. Wang, Y. Yang, *Chinese Chemical Letters* 2024, *35* (10), 109572.

[19]	R. Han, X. Ma, J. Wang, B. Zhang, M. Ruan, J. Jiao, W. Zhao, J. Zhang, *Sensors and Actuators B: Chemical* 2025, *423*, 136766.

[20]	J. Chen, C. Shao, X. Wang, J. Gu, H.-L. Zhu, Y. Qian, *Chemical Communications* 2020, *56* (27), 3871.

[21]	L. Fan, R. Wang, Q. Zan, K. Zhao, Y. Zhang, Y. Huang, X. Yu, Y. Yang, W. Lu, S. Shuang, X. Yang, C. Dong, *Chemical & Biomedical Imaging* 2025, *3* (5), 332.

[22]	C. Shao, Y. Liu, Z. Chen, Y. Qin, X. Wang, X. Wang, C. Yan, H.-L. Zhu, J. Zhao, Y. Qian, *Cell Chemical Biology* 2022, *29* (1), 43.

[23]	Y. Yang, Y. Zhang, M. Ma, H. Liu, K. Ge, C. Zhang, M. Jin, D. Liu, S. Wang, C. Yin, J. Zhang, *Analytical Chemistry* 2022, *94* (41), 14443.

[24]	C. Shao, J. Yuan, Y. Liu, Y. Qin, X. Wang, J. Gu, G. Chen, B. Zhang, H.-K. Liu, J. Zhao, H.-L. Zhu, Y. Qian, *Proceedings of the National Academy of Sciences* 2020, *117* (19), 10155.

[25]	R. Zhang, J. Zhao, G. Han, Z. Liu, C. Liu, C. Zhang, B. Liu, C. Jiang, R. Liu, T. Zhao, M.-Y. Han, Z. Zhang, *Journal of the American Chemical Society* 2016, *138* (11), 3769.

[26]	Q. He, S. Zang, Y. Zeng, B. Wang, X. Song, *Spectrochimica Acta Part A: Molecular and Biomolecular Spectroscopy* 2025, *328*, 125464.

[27]	T. Yu, X. Zhang, Y. Li, X. Zhang, Y. Zhang, H. Li, Y. Deng, P. Yin, S. Yao, *Chemical & Biomedical Imaging* 2025, *3* (10), 691.

[28]	J. Han, X. Liu, H. Xiong, J. Wang, B. Wang, X. Song, W. Wang, *Analytical Chemistry* 2020, *92* (7), 5134.

[29]	H. R. Bolland, E. M. Hammond, A. C. Sedgwick, *Chemical Communications* 2022, *58* (76), 10699.

[30]	L. Wu, J. Liu, X. Tian, R. R. Groleau, S. D. Bull, P. Li, B. Tang, T. D. James, *Chemical Science* 2021, *12* (11), 3921.

[31]	T. Huang, S. Yan, Y. Yu, Y. Xue, Y. Yu, C. Han, *Analytical Chemistry* 2022, *94* (2), 1415.

[32]	X. Bao, W. Shi, Y. Cui, J. Zhang, B. Liang, B. Zhou, C. Huo, *Sensors and Actuators B: Chemical* 2026, *451*, 139362.

[33]	W. Huang, X. Du, C. Zhang, S. Zhang, J. Zhang, X.-F. Yang, *Analytical Chemistry* 2022, *94* (50), 17485.

[34]	Y. Li, J. Cao, X. Wu, J. Kou, T. Feng, R. Zhang, C. Xu, F. Kong, B. Tang, *Analytical Chemistry* 2024, *96* (18), 7138.

[35]	C. Liu, T. Yan, X. Cai, H. Zhu, P. Zhang, X. Liu, X. Rong, K. Wang, Y. Wang, W. Shu, B. Zhu, *Talanta* 2025, *285*, 127408.

[36]	J. Tang, S. Deng, Y. Xie, S. Li, Y. Qiao, L. Zhu, J. Guo, J. Zhou, Y. Ye, *Dyes and Pigments* 2024, *227*, 112183.

[37]	Y. Zhou, J. Zeng, Q. Yang, L. Zhou, *Spectrochimica Acta Part A: Molecular and Biomolecular Spectroscopy* 2022, *282*, 121691.
